# Exploring the potential of laser desorption ionisation time-of-flight mass spectrometry to analyse organic capping agents on inorganic nanoparticle surfaces

**DOI:** 10.1007/s00216-020-02740-3

**Published:** 2020-06-16

**Authors:** Konstantinos Giannopoulos, Oliver J. Lechtenfeld, Timothy R. Holbrook, Thorsten Reemtsma, Stephan Wagner

**Affiliations:** 1 Department of Analytical Chemistry, Helmholtz Centre for Environmental Research – UFZ, Permoserstraße. 15, 04318 Leipzig, Germany; 2 Institute of Analytical Chemistry, University of Leipzig, Linnéstraße 3, 04103 Leipzig, Germany

**Keywords:** Nanoparticle surface, Engineered coating, Characterisation, Surface ligands

## Abstract

Analytical techniques are in high demand for the determination of organic capping agents on surfaces of metallic nanoparticles (NPs) such as gold (Au) and silver (Ag). In this study, the potential of laser desorption ionisation time-of-flight mass spectrometry (LDI-ToF-MS) as a technique fit for this purpose is demonstrated. First, a collection of reference spectra of most commonly used organic capping agents, including small molecules and polymers was established. Second, the robustness of the method was tested towards parameters like NP core material and NP size. In a third step, the quantitative capabilities of LDI-ToF-MS were determined. Finally, the potential to detect chemical alterations of the organic capping agent was evaluated. LDI-ToF-MS is able to detect capping agents ranging from small molecules (citric acid, tannic acid, lipoic acid) to large polymers (polyvinylpyrrolidone, branched polyethylenimine and methoxy polyethylene glycol sulfhydryl) on Au and Ag NPs based on characteristic signals for each capping agent. Small molecules showed characteristic fragment ions with low intensities, whereas polymers showed intense signals of the monomeric subunit. The NP concentration range comprises about two orders of magnitude with lowest detection limits of 5 mg/L or a capping agent concentration in the lower nM range. Changes in capping agent composition are detectable at NP concentrations in the g/L range. Thus, LDI-ToF-MS is particularly suitable for characterisation of polymer-capped NPs with high NP concentrations. This may be the case for quality control as part of the material synthesis and testing.

**Electronic supplementary material:**

The online version of this article (10.1007/s00216-020-02740-3) contains supplementary material, which is available to authorized users.

**Figure:**
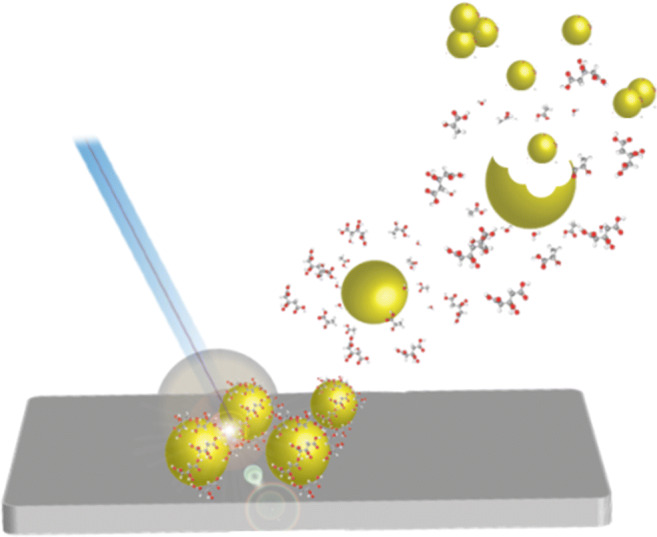
Graphical abstract

## Introduction

Nanoparticles (NPs) are commonly produced having an organic capping agent at their surface, such as small molecules or polymers [1] to increase colloidal stability or dispersion [2]. Hence, the capping agent affects the reactivity, transport and transformation of NPs in the environment and in organisms. For instance, aggregation and dispersion of pristine silver (Ag) NPs suspended in natural organic matter (NOM) solution [3] and dissolution of zinc oxide NPs in soil [4] are highly capping agent dependent. Information on organic capping agents is relevant in the context of material development and synthesis, risk assessment [5, 6] and biomedical applications [7]. The determination of their chemical identity is often required by regulations [8].

The direct chemical analysis of the capping agent, however, is not yet state of the art. Several surface-sensitive analytical techniques allow obtaining chemical, molecular and morphological information from the particle surface [9–11] (and citations therein). Among them, laser desorption ionisation time-of-flight mass spectrometry (LDI-ToF-MS) can be utilised to analyse capping agents of NPs (also referred to as surface ligands) because the core material of most metal NPs efficiently absorbs the laser energy at commonly used laser wavelengths (e.g. Nd:YAG laser with 355 nm) and ionizes the surface ligands. Contrary to matrix-assisted LDI (MALDI), LDI does not rely on an organic matrix; hence, no effect of the organic matrix on the ionisation and spectral interferences can be expected [12].

Using ToF as a mass analyser for the ionized ligands has the advantages of high-speed analysis, high transmission, parallel detection of all species and theoretically unlimited mass range [13]. The identification of ions is based on the mass-to-charge (*m/z*) ratio by which a molecular formula can be assigned. During ionisation of the analyte molecule, fragment molecules may be formed resulting in spectra with a signal at the initial *m/z* ratio and the *m/z* ratios of the fragments. The capping agent of NPs can be characterised with LDI-ToF-MS as it has been demonstrated earlier by Yan et al. for organic monolayers on Au NP surfaces [14]. Covalently and coordinatively bound organic compounds on magnetic NP surfaces were also analysed by LDI-ToF-MS [15]. These developments revealed that LDI-ToF-MS can detect positively, negatively and neutrally charged ligands on Au NP surfaces and provide semi-quantitative information on the composition of mixed monolayers of these ligands [14].

The potential of LDI-ToF-MS to detect and quantify engineered capping agents at NP surfaces may be high if the mass spectrum of the capping agent allows identifying the compound. However, significant fragmentation was already observed in surface-assisted LDI (SALDI) applications [16], where NPs instead of an organic matrix are used for the ionisation of analytes. These fragment ions may differ from those obtained by MALDI. For instance, spectra of polyethylene glycol (PEG) exhibited the intact polymer ionisation in the presence of a MALDI matrix but not when attached to Au NPs [17, 18]. Thus, the identified signals obtained by MALDI, SALDI or even another ionisation technique such as electrospray ionisation (ESI) for the same compound might not be readily transferrable if it is directly attached to NPs. Therefore, a systematic study is required to investigate the potential of LDI-ToF-MS. Still, it remains unknown for what type of capping agents the technique provides reliable and robust data and how NP concentration, NP core material and NP size affect the detection.

To explore the potential of this technique, we tested capping agents such as the small molecules as citric acid (CA) and large polymers as polyvinylpyrrolidone (PVP) which are frequently applied. We detected characteristic signals in the *m/z* spectra of the selected organic capping agents and explored the range of detectable organic capping agents with differences in molecular size and binding mechanisms between NP and capping agent. We also determined the effect of NP core material, NP concentration and NP size on the signal intensity, and evaluated the capability to detect changes of the organic capping agent due to the presence of dissolved organic molecules in suspension. Having such a method at hand, considerable progress may become possible in capping agent characterisation with respect to material synthesis, risk assessment and regulation.

## Materials and methods

### Nanoparticles and chemicals

Suspension of 60 nm CA-capped Au NPs was purchased from BBI solutions (Cardiff, UK). All remaining 30, 60 and 100 nm Au NPs and Ag NP suspensions were purchased from nanoComposix (Prague, CZ). Au NP concentration of the suspension was usually 50 mg/L. Ag NP concentration was 20 mg/L, except 60 nm tannic acid–capped Ag NPs which were provided at a stock concentration of 1 g/L. All NPs are suspended in water except of CA-capped Ag NPs which are provided in 2 mM sodium citrate.

Citric acid monohydrate (for analysis) was purchased from Merck (Darmstadt, Germany), and polyvinylpyrrolidone 40 kDa was purchased from Sigma-Aldrich (St. Louis, MO, USA).

### Purification of the stock suspension

To remove free and excess capping agents from the NP suspensions, 2 mL of stock NPs were bath sonicated with 35 kHz for 3 min (Bandelin Sonorex Digitec DT 1028 CH, Berlin, Germany), vortexed for 1 min at a speed of 2850 rpm (Digital Vortex-Genie 2, Scientific Industries, Inc., New York, USA) and then centrifuged at 15,000 rpm for 10 min at 4 °C in a 2-mL Eppendorf Safe-Lock tube using a bench-top centrifuge (Eppendorf 5424 R, Eppendorf AG, Wesseling-Berzdorf, Germany). After centrifugation, the supernatant was removed with a needle syringe and the remaining pellet was redispersed and diluted to its initial stock concentration by adding 2 mL of Milli-Q water (Milli-Q, Integral 5, Merck, Darmstadt, Germany). The volume of the pellet was estimated to be ~ 20 μL. Hence, the concentration in the pellet was 100-fold higher than in the stock suspensions (i.e. for Au NPs = 5 g/L and for Ag NPs = 2 g/L).

### Sample preparation for LDI-ToF-MS analysis

The purified NP suspensions were ultra-sonicated with a VialTweeter set at 190 W whereby ~ 20 W was applied on the sample for 5 s (UP200St, Hielscher Ultrasound Technology, Teltow, Germany) to detach NPs from the tube wall and disintegrate aggregates. For the analysis of the concentration dependence, the pellet was diluted with Milli-Q water to 500, 250, 100, 50, 25, 5, 0.5 and 0.1 mg/L. For size dependence analysis, the pellets of 30-, 60- and 100-nm m-PEG-SH-capped Au NPs were diluted with 2 mL of Milli-Q water to 50 mg/L. For the sorption experiments, the pellet of 60-nm m-PEG-SH-capped Au NPs was diluted with 2 mL of Milli-Q water to 50 mg/L. Of these purified NP suspensions, 1 mL was mixed separately with 1 mL of the solutes (either 3 mg/L PVP or 3 mg/L CA) to obtain a suspension of 2 mL with 25 mg/L Au NPs and 1.5 mg/L of the solute. This suspension was equilibrated for ~ 5 min, centrifuged, redispersed and diluted with 2 mL of Milli-Q water to 25 mg/L Au NPs and again centrifuged. After supernatant removal, the obtained pellet (~ 20 μL with 2.5 g/L) was either redispersed and diluted with Milli-Q water to 100, 50, 25 and 5 mg/L or directly measured. The sample was deposited on a stainless steel target (384 MTP ground steel, Bruker Daltonics, Bremen, Germany) with a volume of 3 × 2.5 μL to ensure complete coverage of the spot. The air-dried spots had circular shapes with a diameter of ~ 2 mm which was subjected to further analysis with LDI-ToF-MS.

### LDI-ToF-MS analysis

Mass spectra for LDI were recorded on a reflectron-type ToF Bruker Autoflex Speed mass spectrometer (Bruker Daltonics, Bremen, Germany) equipped with a 355-nm Nd:YAG laser in positive and negative ion mode. In reflector, positive and negative mode analysis was performed in the mass range of *m/z* 0–2000 and in the linear positive mode in the mass range of *m/z* 2–50,000. Calibration was performed using Au and Ag clusters. The laser energy was set to “ultra” (energy of 20 μJ and 25 μm diameter at the desorption border). The software flexControl version 3.4 (Bruker Daltonics, Bremen, Germany) was used to configure and operate the instrument, while flexAnalysis 3.4 (Bruker Daltonics, Bremen, Germany) was utilised for spectra treatment.

### Qualitative data acquisition and analysis

For qualitative data acquisition of the stock and purified suspensions as well as the sorption experiments, four single spectra with each 500 shots were accumulated to obtain a final averaged spectrum from 2000 shots. The spectra were acquired using random walk in partial mode (50 shots at each raster spot and 2000 μm limit diameter). Laser power was set between 40 and 80%. Characteristic capping agent and metal core signals were assigned with a threshold of signal to noise (S/N) ratio ≥ 5. The following deflection masses were used, as not otherwise stated: CA 180 Da, TA 320 Da, LA 170 Da, PVP 100 Da, m-PEG-SH and BPEI 55 Da (notice that deflection mass is not accurate and can still show masses below the set deflection value).

### Quantitative data acquisition and analysis

For quantitative data acquisition of the concentration and experiments on size dependency, the spectra were automatically recorded with the implemented function “AutoXecute” of the software. The benefit of “AutoXecute” is the possible accumulation of spectra after defining parameters for a selected signal (e.g. resolution, S/N, peak width) using random walk and varying the laser energy after each shot. As a consequence, only spectra are accumulated containing capping agent signals, and thus, the final averaged spectrum is not falsified by the addition of spectra without capping agent signals. This allows a quantitative comparison of the capping agent intensities ensuring robustness, spot-to-spot and shot-to-shot reproducibility. Moreover, the sample was spotted on the target in six replicates and each was measured three times resulting in 18 measurements per sample.

For each measurement, 10 mass spectra from 200 laser shots each were accumulated to obtain a final averaged spectrum with 2000 shots. The 200 laser shots were accumulated in 4 × 50 laser shots. The spectra were acquired using random walk. Laser power was set between 40 and 60%. For signal identification, the mass range was set either for the monomer as for m-PEG-SH and BPEI (43–47 Da) or for the monomer adduct for PVP (138–139 Da). Further parameters for spectrum analysis were peak resolution: 100; peak detection algorithm: centroid; S/N threshold: 5; minimum intensity threshold: 10; maximum number of peaks: 10; peak width: 0.02 *m/z*; peak height and relative intensity threshold: 0%; baseline subtraction: Top Hat; smoothing: Savitzky-Golay. The LOD was reached if (i) an interference signal was accumulated, (ii) the three measurements of one of the six replicates showed only zero intensity values and (iii) less than 10 mass spectra for each measurement were accumulated. In case of (i) and (ii), the next higher calibration-level was used to conservatively estimate the LOD, whereas in (iii), LOD was equal to this measured concentration.

### Total organic carbon analysis

Total organic carbon (TOC) measurements were performed on a multi N/C 2100 S analyser (Analytik Jena AG, Jena, Germany). The carbon concentration in the samples was determined as non-purgeable organic carbon (NPOC) with triplicate injection (100 μL). The analysed samples of the supernatants and the stock and purified suspensions were measured undiluted in triplicates.

## Results and discussion

A set of six different organic capping agents on Au NPs as well as on Ag NPs was used to explore the capability of LDI-ToF-MS analysing organic capping agents of NPs (Table 1). These commercially available and frequently applied capping agents cover a broad range of possible interactions between organic molecules and metal NP cores from weak electrostatic interaction (CA) over metal-to-ligand charge transfer (MLCT) and van der Waals (vdW) forces (TA and PVP) to strong covalently bound (LA, BPEI and m-PEG-SH). A wide range of molecular weights is used: Small molecules from approx. 200 Da (CA, LA) to 1700 Da (TA) as well as large polymers from 5 kDa (m-PEG-SH) to 40 kDa (PVP). These capping agents varied also in surface charge representing neutral, positive and negative molecules (Table 1).

**Table 1:**
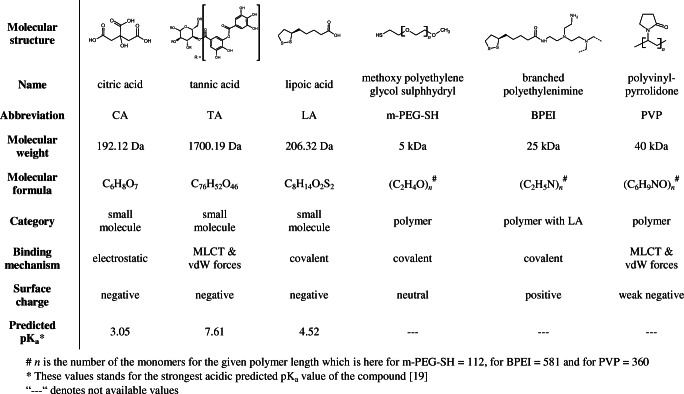
Selected capping agents and their characteristics for the investigation of LDI-ToF-MS

^#^*n* is the number of the monomers for the given polymer length which is here for m-PEG-SH = 112, for BPEI = 581 and for PVP = 360

*These values stands for the strongest acidic predicted pK_a_ value of the compound [19]

“---” denotes not available values

**Fig. 1:**
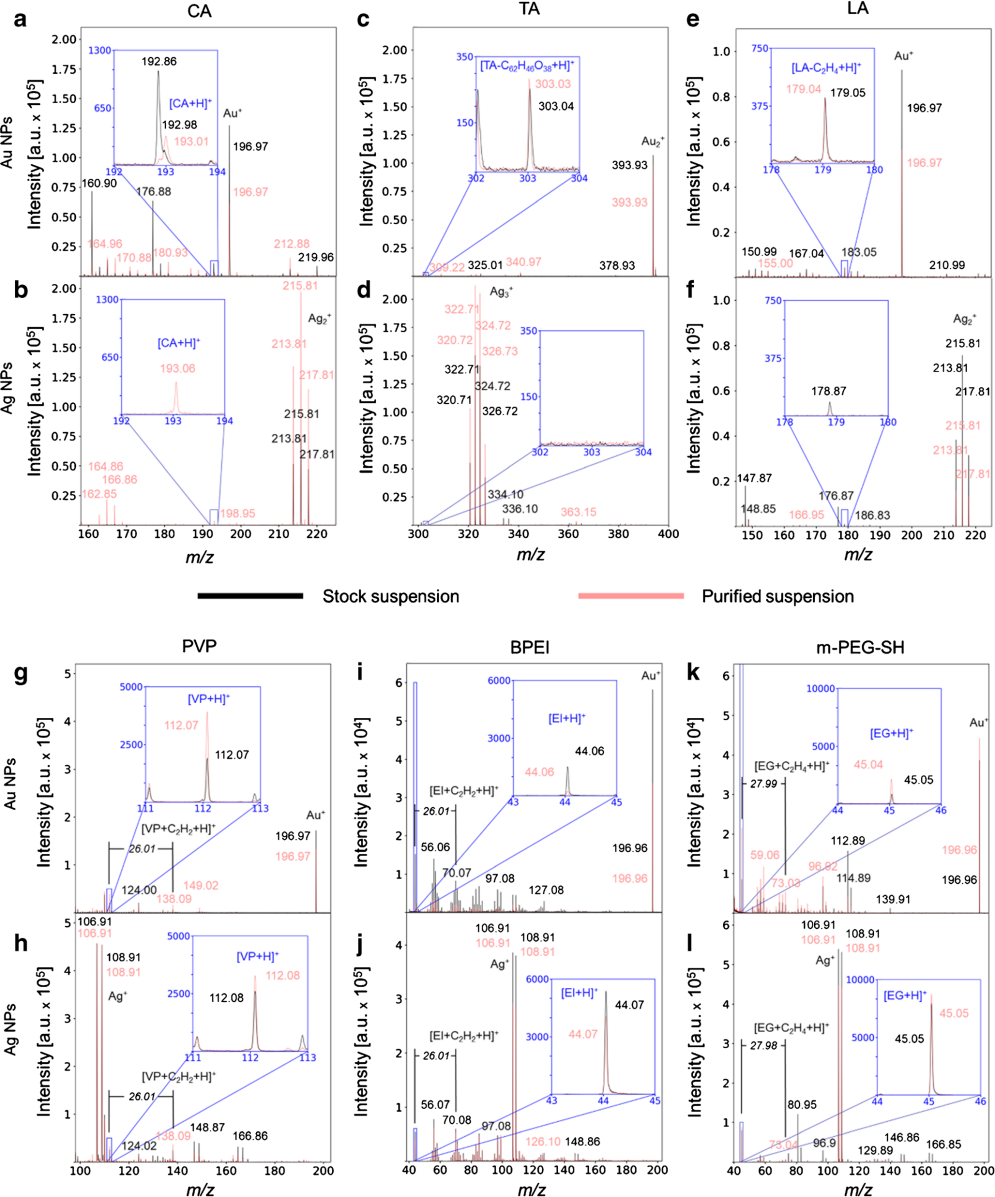
Spectra of the stock and purified suspensions for Au NPs (upper spectrum) and Ag NPs (lower spectrum) with organic capping agents of CA (**a**, **b**), TA (**c**, **d**), LA (**e**, **f**), PVP (**g**, **h**), BPEI (**i**, **j**) and m-PEG-SH (**k**, **l**) in the positive mode whereby the red line represents the stock suspension and the black line the purified suspension

Three criteria were applied to identify characteristic capping agent signals in the mass spectra: (1) a signal appears at the *m/z* value of the molecular ion or a reasonable fragment ion, (2) signals have been previously reported in literature and (3) the S/N ratio exceeds 5. For the small molecules, we aimed to detect the molecular ions. For the polymers, we aimed at the intact polymer chains in a mass range corresponding to its mean molecular weight (up to 40 kDa) or at characteristic fragment ions. The overlaid spectra of the stock and purified suspension for a direct visual comparison are depicted in Fig. 1 in which only a section of the *m/z* range is shown in which the characteristic signal was found. The raw spectra with the entire acquired *m/z* range are given in the Electronic Supplementary Material (ESM) in Figs. S1–S37. In Table 2, all identified characteristic ions are listed from the stock and purified suspension together with their S/N ratios which can be used for relative comparison since the same instrumental conditions for spectra acquisition were applied. The calculated mass errors are shown in ESM Table S1.

**Table 2:** Summary of the identified characteristic signals with formula and exact mass for each organic capping agent on Au and Ag NPs in the stock and purified suspension in the positive mode

Capping agent	Detected ion	Exact mass [*m/z*]	S/N ratios			
			Au NPs		Ag NPs	
			Stock	Purified	Stock	Purified
CA	[CA + H]^+^	193.034	17*	28	---	35
TA^§^	[TA-C_62_H_46_O_38_ + H]^+^	303.014	32	32	---	---
LA	[LA-C_2_H_4_ + H]^+^	179.019	36	37	---	---
PVP	[VP + H]^+^	112.076	190	263	104	118
	[VP + C_2_H_2_ + H]^+^	138.091^#^	74	122	118	146
BPEI	[EI + H]^+^	44.049	239	100	617	559
	[EI + C_2_H_2_ + H]^+^	70.065^#^	113	63	250	237
m-PEG-SH	[EG + H]^+^	45.033	161	259	1356	1432
	[EG + C_2_H_4_ + H]^+^	73.065^#^	63	114	90	94

*This value for Au-CA in the stock suspension was manually readout due to the overlapping signal at *m/z* 192.86

^#^These additional identified polymeric fragment ions are referred to as “monomer+C_2_”

^§^The TA fragment ion was also detected as the corresponding anion on Au NPs

“---” denotes not assigned signals due to S/*N* < 5

### LDI-ToF-MS analyses of stock suspensions

#### Small molecule capping agents

The mass spectra of the small molecules showed for CA on Au NPs in the positive mode the molecular ion [CA + H]^+^ at *m/z* 192.98 as a shoulder of an overlapping signal at *m/z* 192.86 (Fig. 1a). However, on Ag NPs, neither the [CA + H]^+^ ion was detected (Fig. 1b) nor previously reported sodium adducts [CA + Na]^+^ or [CA-H + 2Na]^+^ [20] which could be formed since these NPs are dispersed in 2 mM sodium citrate.

For the other two small molecules TA and LA, the molecular ions were not detected neither for Au NPs nor for Ag NPs. Instead, fragment ions appeared in the mass spectra. TA on Au NPs was identified by a fragment ion in positive mode at *m/z* 303.04 (Fig. 1c). This fragment ion can be assigned to ellagic acid (EA) with the molecular formula C_14_H_6_O_8_. The existence of EA is further supported by a signal in the negative mode at *m/z* 301.01 which represents the corresponding anion (ESM Fig. S38a). For TA on Ag NPs, neither this fragment ion (Fig. 1d) nor other characteristic fragment ions could be detected.

In case of LA on Au NPs, the signal at *m/z* 179.05 in positive mode (Fig. 1e) may originate from the neutral loss of ethylene as [LA-C_2_H_4_ + H]^+^. For LA on Ag NPs, neither this fragment ion nor other characteristic ions were assigned in the positive mode. Nevertheless, in the negative mode, the presence of LA is indirectly confirmed by the presence of metal-sulfur cluster ions detected as [Au_*n*_S]^−^ and [Ag_*n*_S]^−^ with *n* = 1–3 (ESM Fig. S39). These metal-sulfur cluster ions might be formed from the covalent bonds of LA via two thiol groups (also called sulfhydryls) to either Au or Ag. The calculated binding energy of the thiol-Au bond is ~ 44 kcal/mol and is much higher compared with the carboxylic acid-Au bond with only ~ 2 kcal/mol as present in CA [21] (and citations therein). It appears that this high binding energy hinders the fragmentation of the S-Au bond, while the S-C bond is cleaved first, leading to the subsequent formation of clusters of Au and S [22]. Moreover, the large sulfur atom is capable of stabilizing the negative charge of these fragment anions.

Our identified signals for the small molecules used here are not reported in literature with LDI-MS. However, our observed fragment ions for CA, TA and LA have been identified as characteristic mass spectrometry fragments for these compounds [20, 23–25]. However, the ion formation is not only solely dependent on the type of capping agent but also on the core material. Furthermore, matrix constituents such as salts may affect the ionisation and, thus, the mass spectrometric detection.

#### Polymeric capping agents

The characteristic pattern of the mass signals typical for polymers was not detected by LDI-ToF-MS for the three tested polymeric capping agents. Instead, signals of the respective monomers were detected for both metals in positive mode: (i) PVP as [VP + H]^+^ at *m/z* 112.07 (Fig. 1g, h); (ii) BPEI as [EI + H]^+^ at *m/z* 44.06 (Fig. 1i, j); and (iii) m-PEG-SH as [EG + H]^+^ at *m/z* 45.05 (Fig. 1k, l). For each of the three capping agents, a second fragment ion was identified. These fragment ions are the monomers carrying an additional C_2_H_2_ group for PVP and BPEI, while for m-PEG-SH, a C_2_H_4_ group at *m/z* 138.09, 70.07 and 73.04, respectively, and are thus from now on referred to as “monomer+C_2_”. The intensity of these fragment ions will be compared with the intensity of monomer ions in the experiments on concentration and size dependence. The sulfur linker of BPEI and m-PEG-SH were visible in the negative mode as Au-S and Ag-S clusters, similar to LA (ESM Figs. S40 and S41).

Of the three polymer compounds, only PEG has been investigated on NPs before. Chang et al. showed that no polymeric PEG signal was observed for different functional PEG-modified Au NPs with a molecular weight of 5 kDa but only in the presence of HgTe nanomatrices [18]. This is in agreement with our results as we did not observe signals at the *m/z* ratios in the mass range of the intact polymer chains. MALDI analysis of polymers showed that it was not possible to obtain intact ions of the polymer either. For instance, PEI with a molecular weight of ~ 8 kDa was not detected by its molecular ions but showed fragment ions in the range *m/z* 200–1000 [26]. A study of 20 kDa PVP revealed only fragment ions up to *m/z* 5000 [27], whereas in another study, a polydisperse 40 kDa PVP did not allow to obtain structural information such as the repeating unit or the end groups [28].

In comparison with the characteristic signals of the small molecules, the monomers of the respective polymers show higher relative intensities and S/N ratios (Fig. 1, Table 2) which facilitate their identification. This is because many identical monomeric ions may be formed from one polymeric molecule which in turn leads to a high signal intensity of the monomeric ion.

### LDI-ToF-MS analyses of purified NP suspensions

The stock suspensions under study contain suspended capped NPs and dissolved constituents such as organics and salts which remain in suspension after synthesis. Therefore, NP suspensions were purified by centrifugation and subsequent resuspension in Milli-Q water. For weakly bound capping agents, however, such a procedure may lead to the loss of the capping agent. Details on the purification procedure and the quality control measurements are provided in the ESM in Table S2.

#### Small molecule capping agents

The LDI-MS spectra after purification compared well with those of the stock suspension for small molecules attached to Au NPs. Regarding the binding mechanisms of the small molecules, CA is weakly electrostatically bound to the surface via carboxylic acid groups. In contrast to our expectations, CA was still visible in the spectra after purification (Fig. 1a): it has been reported that carboxylates increase their binding energy with decreasing surface coverage up to 55 kcal, thus preventing complete detachment [29]. Also, the polyphenolic TA remained visible: obviously, its interaction with the Au NPs by MLCT and vdW forces was strong enough to prevent its loss during purification (Fig. 1c, ESM Fig. S38b). As expected, the two strong covalent bonds of LA to the Au NPs allowed the identification of the LA fragment ion (Fig. 1e). In contrary, analysis of the purified Ag NP suspensions with the tested capping agents revealed differences compared with the stock suspensions. A signal for CA at *m/z* 193.06 appeared which was not present in the stock suspension (Fig. 1b). This suggests that the 2 mM of sodium citrate in the stock suspension may have suppressed the ionisation of CA. To test this hypothesis, the stock suspension was analysed after on-spot washing with Milli-Q water on the target. After three consecutive washing steps, the protonated CA ion [CA+H]^+^ was detected (data not shown). For TA on Ag NPs after purification, neither the assigned expected fragment ion of EA on Au NPs nor other characteristic ions were observed (Fig. 1d). However, the TOC measurements supported a stronger attraction of TA to Au NPs than to Ag NPs (ESM Table S2). For LA on Ag NPs, purification reduced and removed possible contaminants such as at *m/z* 147.87 and 178.87; nevertheless, no molecular ion or characteristic fragment ion of LA was visible (Fig. 1f). However, the metal-sulfur cluster ions in the negative mode were still present (ESM Fig. S39) which further confirms the strong covalent bond between LA and the NP surface.

#### Polymeric capping agents

For all three polymers, the specific signals from the stock suspension were also detected after purification on both metal NPs (Fig. 1g–l). BPEI and m-PEG-SH are strongly covalently bound to the surface, and also the MLCT and vdW forces between PVP and the surface were obviously strong enough to withstand the purification procedure. The Au-S and Ag-S clusters of BPEI and m-PEG-SH were also detected after purification (ESM Figs. S40, S41).

### Concentration dependency of the signal intensity

The quantitative capabilities of LDI-ToF-MS analysis were evaluated for all three polymeric capping agents because of their good detectability after purification. Changes in the monomer signal intensities were determined for NP mass concentrations between 0.1 and 500 mg/L for 60-nm-sized NPs. The concentration of capping agent at the surface is often not known and can only be estimated based on grafting density values. The grafting density value, however, is dependent on the material and the capping agent. Literature values usually vary by orders of magnitude making such an estimation highly uncertain (ESM Table S3). Therefore, we relate our data to the known NP concentration and TOC concentration data.

**Fig. 2:**
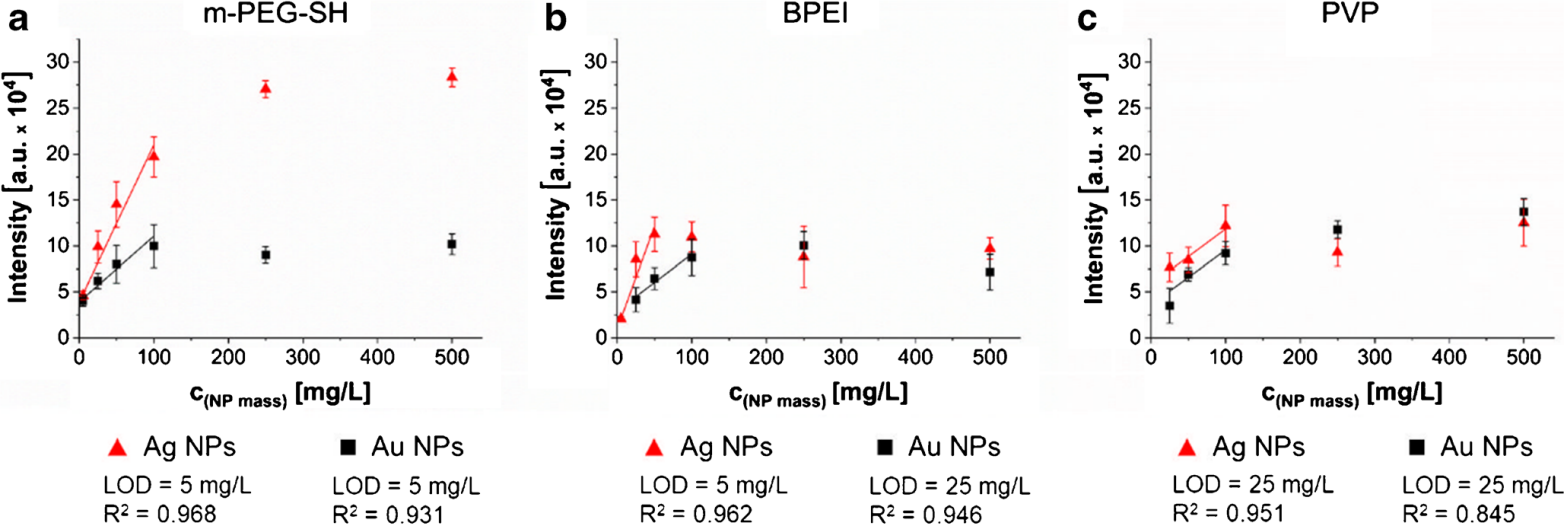
Monomer signal intensity versus NP mass concentration of 60 nm Au and Ag NPs capped with **a** m-PEG-SH, **b** BPEI and **c** PVP. The solid line depicts the linear regression. Error bars were determined from three measurements of six spot replicates (*n* = 18)

The intensities of the monomeric ions for all tested polymers increased linearly with increasing NP concentration over two orders of magnitude (Fig. 2) before the saturation limit was reached at approx. 100 mg/L (Fig. 2a–c). The limit of detection (LOD) was dependent on the type of capping agent and ranged from 5 mg/L for NPs capped with m-PEG-SH to 25 mg/L for PVP. The detectable NP mass concentration range (5–500 mg/L) corresponds to a range of surface areas between 0.3 and 48 × 10^17^ nm^2^ (ESM Fig. S42). This corresponds to LODs for the capping agents in the lower nM range (Table 3, details in ESM, Section 3).

**Table 3:** Capping agent concentration in [nM] of the three polymeric capping agents on Au and Ag NPs estimated from the TOC concentration. LOD values were specified according to the criteria listed in the method section

NPs	Capping agent	Unit	NP mass concentration (mg/L)					
			5	25	50	100	250	500
Au	PVP	[nM]	---	24*^#^	*48*	*95*	239	477
	m-PEG-SH		37*	*183*	*367*	*734*	1834	3668
	BPEI		---	36*	*72*	*143*	359	717
Ag	PVP	[nM]	---	48*^#^	*95*	*191*	477	954
	m-PEG-SH		73*	*367*	*734*	*1467*	3668	7335
	BPEI		14*	*72*	*143*	287	717	1434

The italicised table entries are concentrations representing the linear range shown in Fig. 2

*These values represent the LOD for each NP-capping agent combination

^#^For PVP, the monomer+C_2_ was used to identify the LOD

“---” denotes concentrations below LOD

The monomer signal intensity of m-PEG-SH on Ag NPs was approximately three times higher than on Au NPs (Fig. 2a). Furthermore, the plot of the signal intensity ratio of the monomer to monomer+C_2_ ions (ESM Fig. S43) supports the preferential fragmentation to the monomer ion in the presence of Ag NPs due to an increase of the ratio with increasing NP mass concentration. However, for the other NP-capping agent combinations, the intensity ratio remains constant over the tested concentration range revealing consistency in fragmentation within the capping agents. Consistent with our results, in SALDI applications using Au NPs for the detection of aminothiols, LODs were also reported in the nM concentration range together with linearity between LOD and 0.8 μM [30]. Additionally, a saturation trend of signal intensity with increasing concentrations was also observed in SALDI applications [31, 32]. However, in SALDI, saturation may occur when NP surface is completely covered by the respective analyte [31]. In our experiments, we assume that the plateau is formed due to laser saturation, meaning that no more capping agent can be ionized with the given laser settings. It was not tested whether signal intensities change at higher and lower laser energy settings. Ng et al. showed that with increasing laser fluence, the ion-desorption efficiency is increased [33], i.e. higher sensitivity can be reached. However, it needs to be elucidated how increased laser energy affect the fragmentation of the capping agents.

### NP size dependency of the signal intensity

**Fig. 3:**
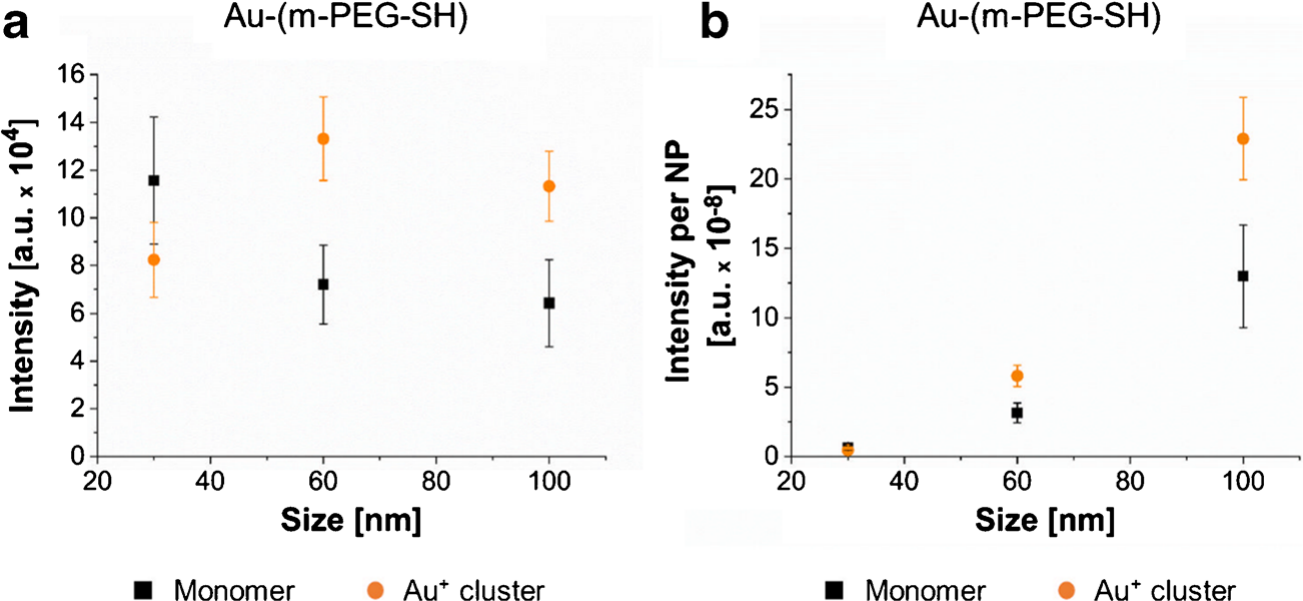
Monomer and Au^+^ cluster ions signal intensity versus NP size of 30-, 60- and 100-nm m-PEG-SH-capped Au NPs. **a** Absolute intensity of the m-PEG-SH monomer and Au^+^ cluster ions. **b** Intensity per NP for the m-PEG-SH monomer and Au^+^ cluster ions. Error bars were determined from three measurements of six spot replicates (*n* = 18)

The effect of NP size on the signal intensity was further explored using m-PEG-SH-capped Au NPs with sizes of 30, 60 and 100 nm. The stock suspensions were purified and diluted to the initial concentration of 50 mg/L. The signal intensities of the monomer and the Au cluster ions were compared for the different NP sizes. The absolute intensity of the monomer signal decreased with increasing NP size (Fig. 3a). This may be explained by a higher surface-to-volume ratio of smaller NPs at the same mass concentration resulting in (i) higher total number of NPs, (ii) higher total surface area and (iii) higher total number of attached m-PEG-SH molecules (ESM Table S4). Thus, the increase in signal intensity may originate from the presence of higher amounts of capping agent. In SALDI applications, it is also reported that the highest signal intensities were achieved with smaller sized Au [34, 35] and Ag NPs [36]. The intensity ratios of the monomer and monomer+C_2_ ions (ESM Fig. S44) for 60- and 100-nm-sized NPs were both approximately 2.06 ± 0.41. Only for 30 nm a significantly higher value with 3.34 ± 0.51 was obtained (*p* = < 0.001) indicating that the smaller sized NPs fragment the polymer more to the monomer ion.

The intensity per NP (Fig. 3b) showed the opposite behaviour for the monomeric ion; it increased with increasing NP size. NPs of larger size ionize more efficiently and fragment less because of (i) the larger cross section for photoabsorption [37], (ii) the higher number of m-PEG-SH per NP [38] (ESM Table S4) and (iii) the lower surface energy [39] which results in decreased binding energies and, thus, to an easier removal of the capping agent. These results show the influence of NP size on ionisation and fragmentation. They outline that the capabilities of LDI-ToF-MS for quantitation are limited because the signal is not solely dependent on the number of attached molecules but also on NP size. Regarding the Au cluster ions, increased intensities with increasing NP size (Fig. 3a) were also reported for SALDI applications [34]. However, a less pronounced intensity increase was observed for 100 nm which may have been limited due to laser saturation. The intensity of the Au cluster per NP also increased (Fig. 3b) because the NP mass increases with increasing NP size.

### Sorption of organic solutes onto capped NPs

LDI-ToF-MS is suitable to identify engineered capping agents on NP surfaces. In this section, we explore whether it is also applicable to detect sorption onto the capped NPs. As a proof of concept, it was tested under which analytical conditions such changes are detectable with LDI-ToF-MS. For this purpose, 60-nm m-PEG-SH-capped Au NPs were mixed with a solution of 40 kDa PVP as a large polymer and with a solution of CA as a small molecule, respectively. After equilibration, a range of Au NP concentrations was then analysed by LDI-ToF-MS.

**Fig. 4:**
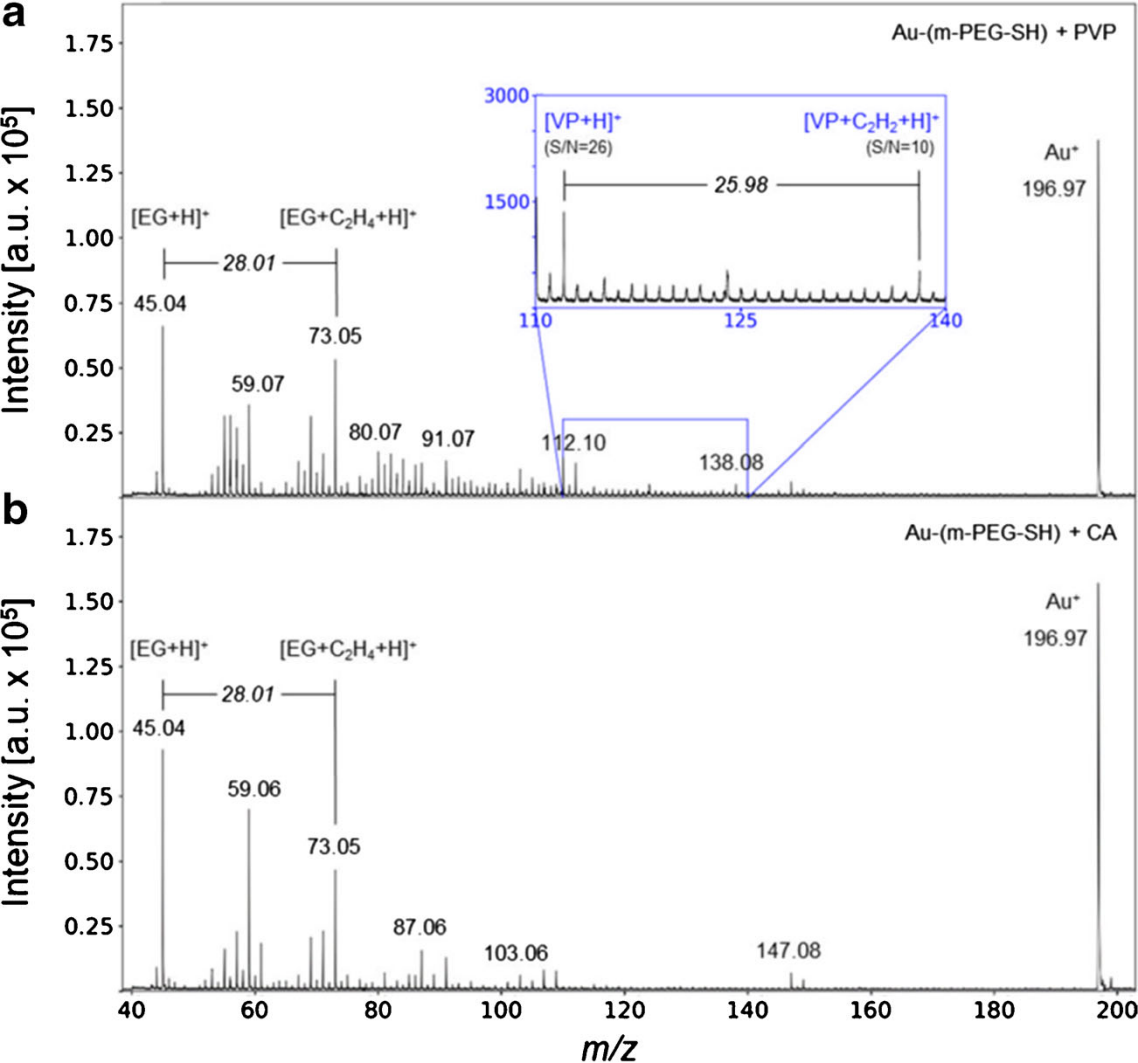
Spectra of sorption experiments of organic solutes onto 60-nm m-PEG-SH-capped Au NPs after interaction with a solution of **a** 40 kDa PVP and **b** CA

The PVP monomer ion [VP + H]^+^ and the monomer+C_2_ ion [VP + C_2_H_2_ + H]^+^ at *m/z* 112.10 and 138.08, respectively, were present in the spectra obtained from a highly concentrated m-PEG-SH-capped Au NP suspension with PVP solution (NP concentration 2.5 g/L) after purification (Fig. 4a). Additionally, the characteristic signals for m-PEG-SH were present in the spectra at *m/z* 45.04 and 73.05, respectively. This indicates that PVP attaches to the m-PEG-SH-capped surface. In case of lower NP concentrations (5 and 100 mg/L), the presence of the initial m-PEG-SH capping agent but not of the PVP was confirmed by LDI-ToF-MS analysis. In contrast, CA mixed with m-PEG-SH-capped Au NPs did not show the protonated CA molecular ion neither in the less concentrated suspensions nor in the highly concentrated pellet (Fig. 4b). This suggests that sorption of CA to the m-PEG-SH-capped surface is too low to be detected.

In general, the highly concentrated NP pellet revealed higher signal intensities of about one order of magnitude higher compared with the stock and purified suspensions (Fig. 1k). This allowed the assignment of additional signals such as the capping agent metal cluster (ESM Fig. S45) and capping agent fragment series (ESM Fig. S46). These two experiments showed that (i) the m-PEG-SH-capped Au NPs displayed different interactions with different types of solutes and (ii) the NP mass concentrations have to be in the g/L range in order to detect changes in the spectrum.

## Conclusion

Surface characterisation of NPs is often required to not only gain insights on NP transport, reactivity and transformation but also for quality control during material synthesis and for regulatory demands, e.g. in REACH regulation. Therefore, the potential of LDI-ToF-MS to analyse organic capping agents on metallic NP surfaces was systematically explored using six commercially available capping agents ranging from small molecules (CA, LA, TA) to large polymers (PVP, m-PEG-SH, BPEI), two inorganic cores as Au and Ag and three different NP sizes (30, 60 and 100 nm).

Our screening approach showed that LDI-ToF-MS is a versatile tool to identify the organic capping agent at the surface of different metallic NPs either through their intact molecular ions or by fragment ions. For the three low molecular weight capping agents, purification may be required to remove disturbing solutes. In contrast, all tested polymers were detectable before and after purification at both metals and characteristic and intense monomer ions were detected. This suggests that LDI-ToF-MS is more suitable to detect polymeric capping agents. Moreover, LDI-ToF-MS reveals qualitative information on the composition of the inorganic NP core material. It might be possible to identify unknown cores of NPs due to highly abundant metal clusters and their characteristic isotopic patterns, whereas for unknown capping agents, ultra-high-resolution MS is recommended.

The detection limit for the polymer capping agents is in the nM range which makes it suitable for characterising organic capping agents in NP suspensions. The sensitivity of this technique is also sufficiently high to detect changes in the capping agent composition at NP concentrations in the g/L range. Four factors were shown to restrict the suitability of LDI-ToF-MS for quantitative analysis: (i) the binding mechanism of the capping agent to the core, where stronger binding and smaller sized molecules lead to less characteristic fragment ions; (ii) the composition of the core material which influences both the ionisation efficiency and the fragment ion formation; (iii) the NP mass concentration which can induce laser saturation and loss of sensitivity above 100 mg/L; and (iv) the NP size which was inversely correlated with signal intensity. Ultimately, LDI-ToF-MS is suitable for polymer capping agent characterisation, e.g. not only for quality control as part of the material synthesis but also for regulatory needs in material registration and testing.

## Electronic supplementary material

ESM 1 (PDF 2.52 mb).
