# Supplementary material for: Exploring the potential of laser desorption ionisation time-of-flight mass spectrometry to analyse organic capping agents on inorganic nanoparticle surfaces
Source: Anal Bioanal Chem. 2020 Jun 16;412(22):5261–71. doi: 10.1007/s00216-020-02740-3 (PMC7387369; doi:10.1007/s00216-020-02740-3)
Supplement: Supplementary file 1 — (PDF 2.52 mb). [file 216_2020_2740_MOESM1_ESM.pdf]

Analytical and Bioanalytical Chemistry

Electronic Supplementary Material

Exploring the potential of laser desorption ionisation time-of-flight mass spectrometry to analyse organic capping agents on inorganic nanoparticle surfaces

Konstantinos Giannopoulos, Oliver J. Lechtenfeld, Timothy R. Holbrook,
Thorsten Reemtsma, Stephan Wagner

1 LDI-ToF-MS analyses of stock and purified suspensions

Au-CA from *m/z* 155-780

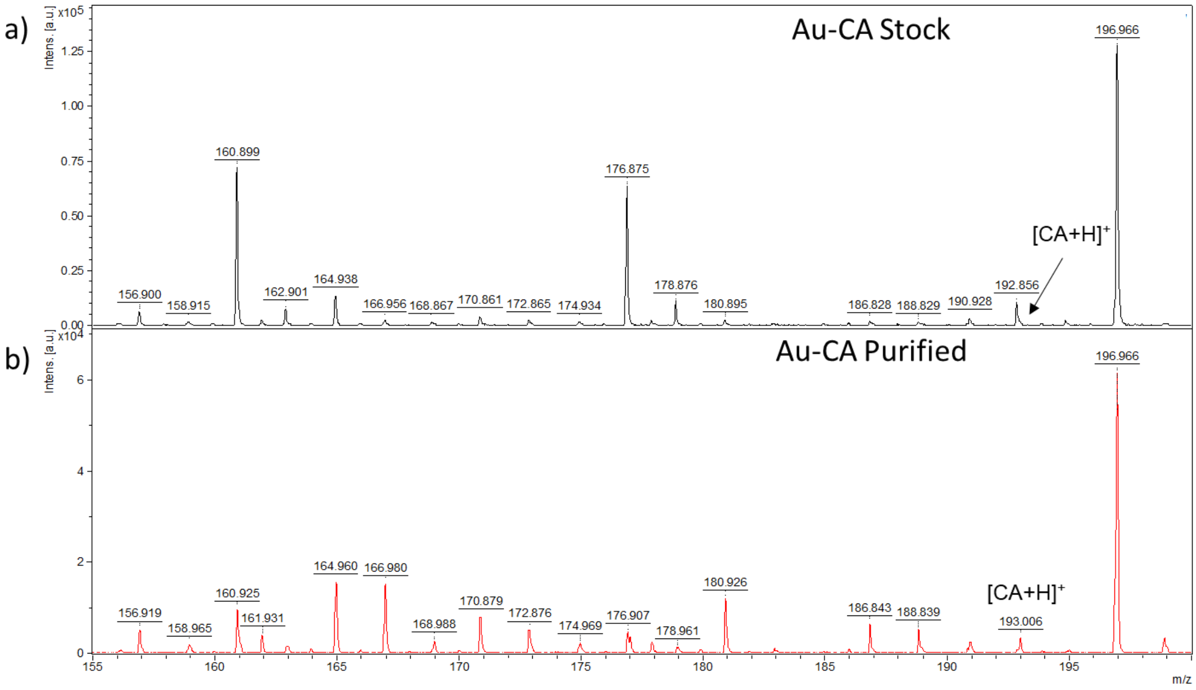

Fig. S1 Raw spectra of CA capped Au NPs in the mass range of *m/z* 155-200 in the positive mode for a) the stock and b) purified suspension

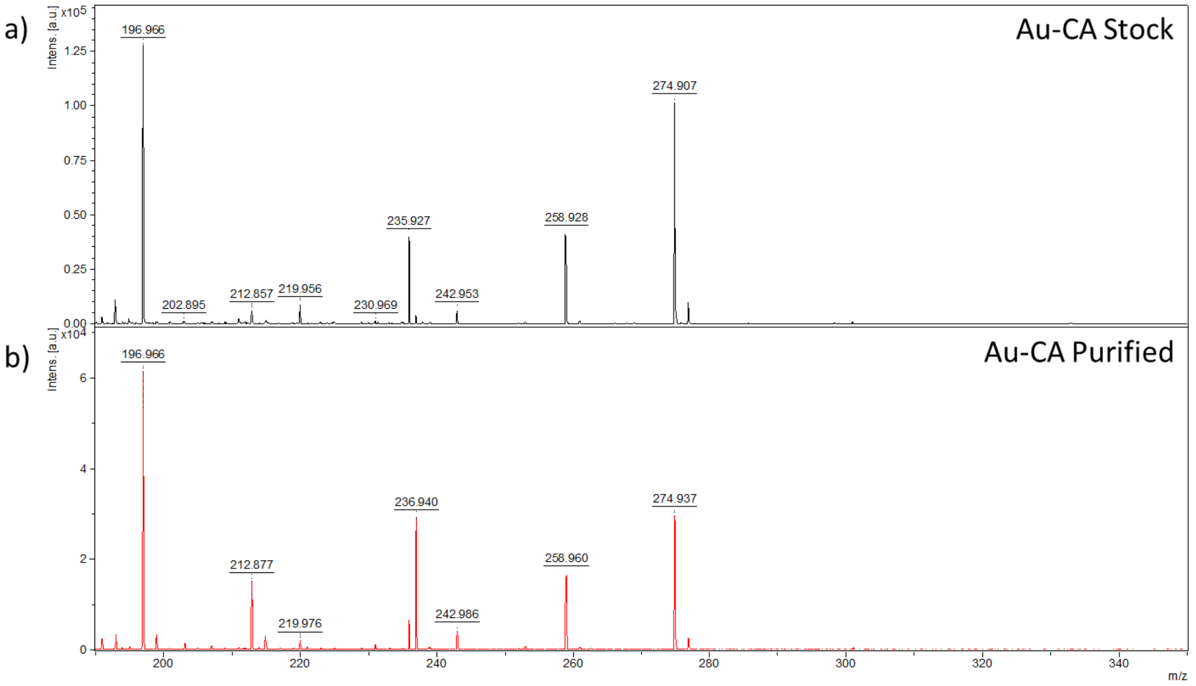

Fig. S2 Raw spectra of CA capped Au NPs in the mass range of *m/z* 190-350 in the positive mode for a) the stock and b) purified suspension

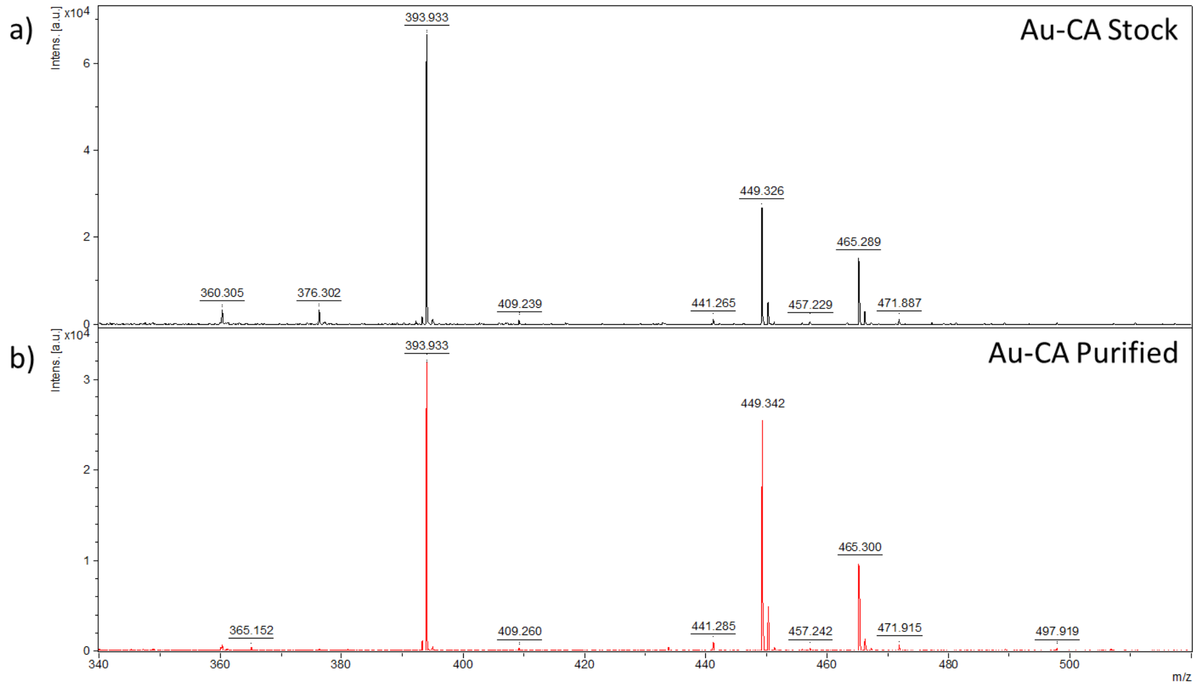

Fig. S3 Raw spectra of CA capped Au NPs in the mass range of *m/z* 340-520 in the positive mode for a) the stock and b) purified suspension

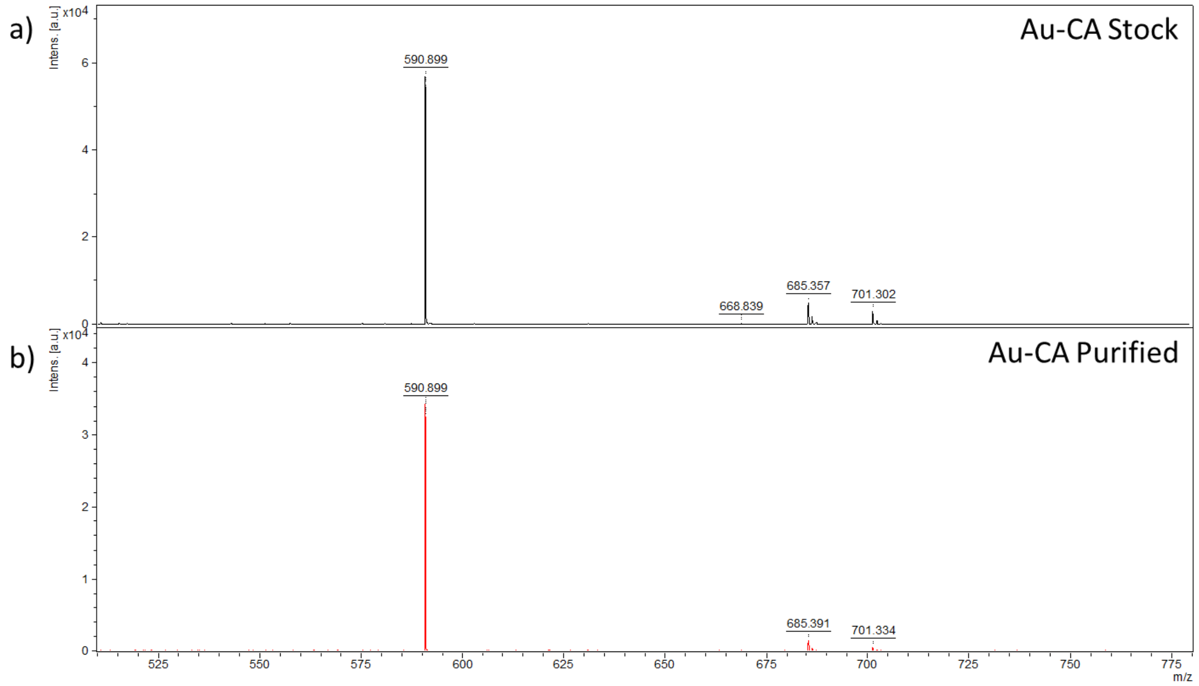

Fig. S4 Raw spectra of CA capped Au NPs in the mass range of *m/z* 510-780 in the positive mode for a) the stock and b) purified suspension

Ag-CA from *m/z* 155-780

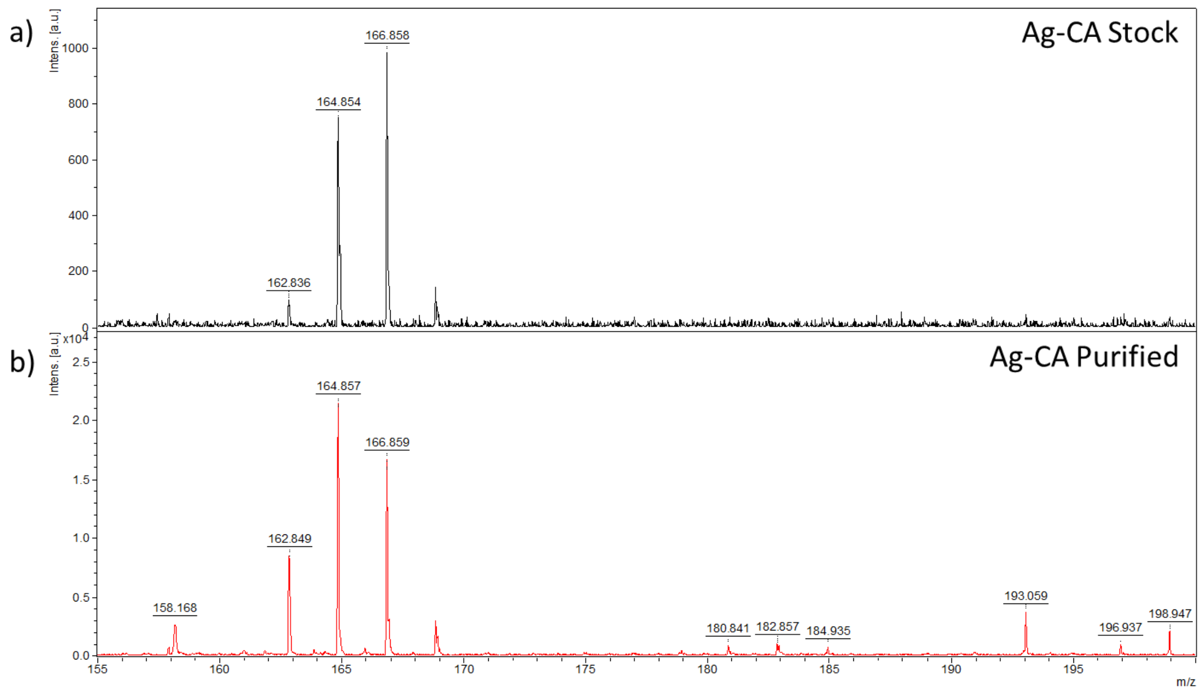

Fig. S5 Raw spectra of CA capped Ag NPs in the mass range of *m/z* 155-200 in the positive mode for a) the stock and b) purified suspension

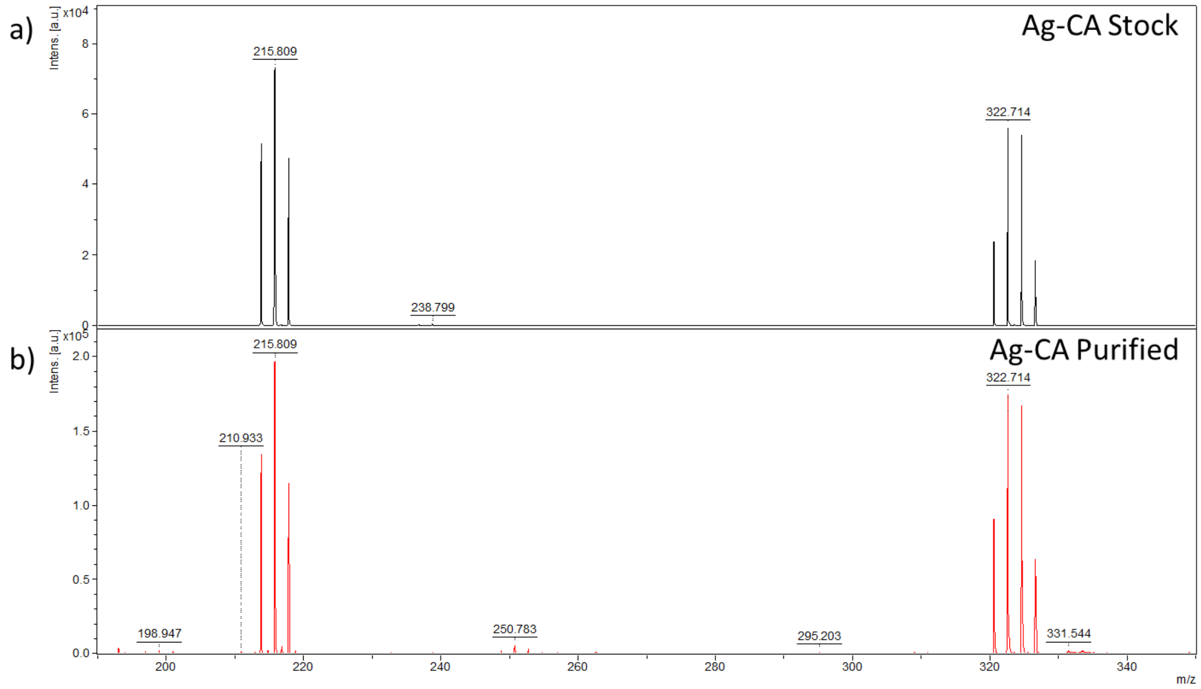

Fig. S6 Raw spectra of CA capped Ag NPs in the mass range of *m/z* 190-350 in the positive mode for a) the stock and b) purified suspension

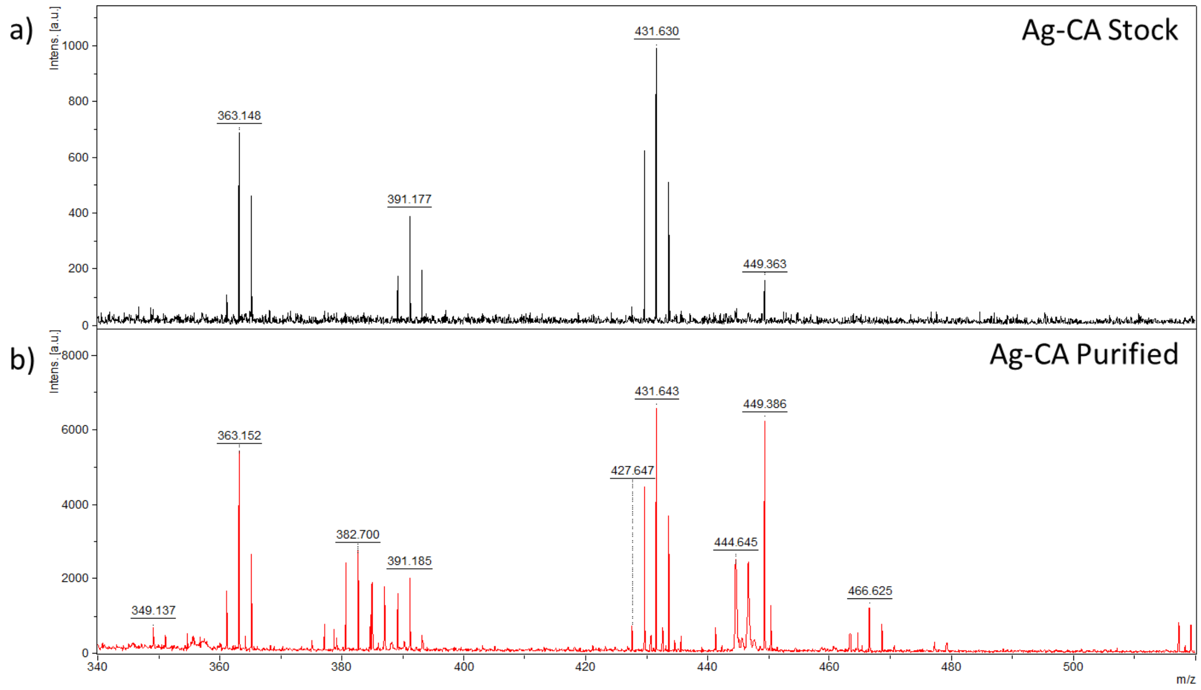

Fig. S7 Raw spectra of CA capped Ag NPs in the mass range of *m/z* 340-520 in the positive mode for a) the stock and b) purified suspension

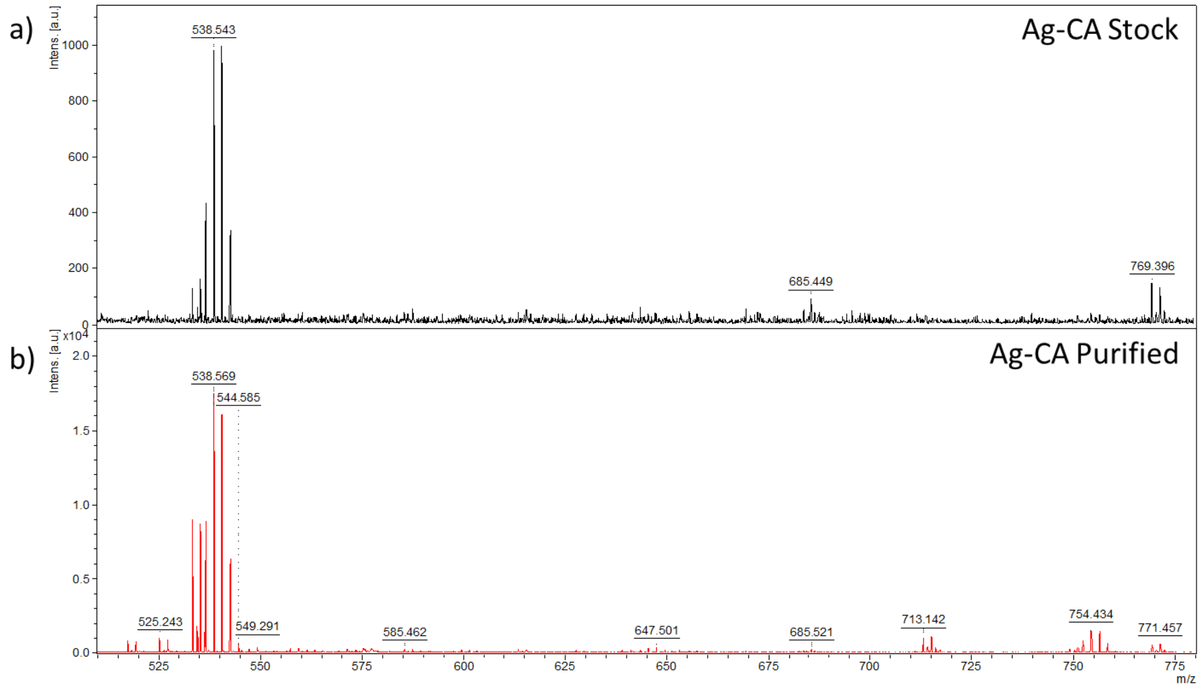

Fig. S8 Raw spectra of CA capped Ag NPs in the mass range of *m/z* 510-780 in the positive mode for a) the stock and b) purified suspension

Au-TA from *m/z* 290-780

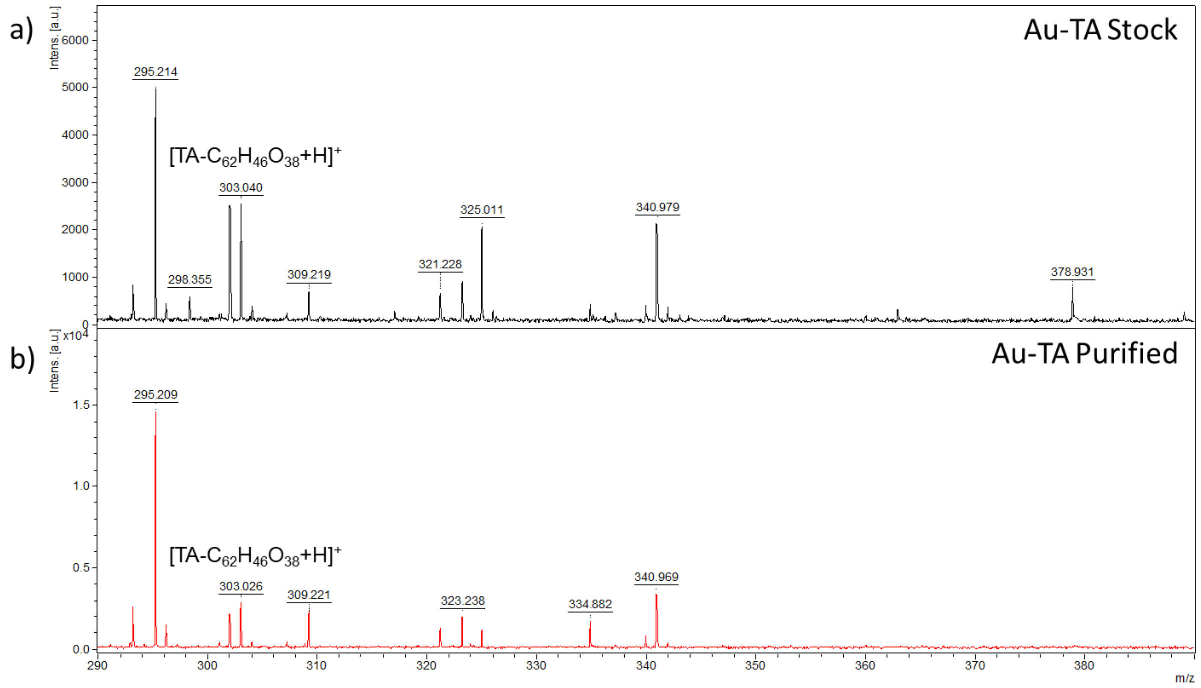

Fig. S9 Raw spectra of TA capped Au NPs in the mass range of *m/z* 290-390 in the positive mode for a) the stock and b) purified suspension

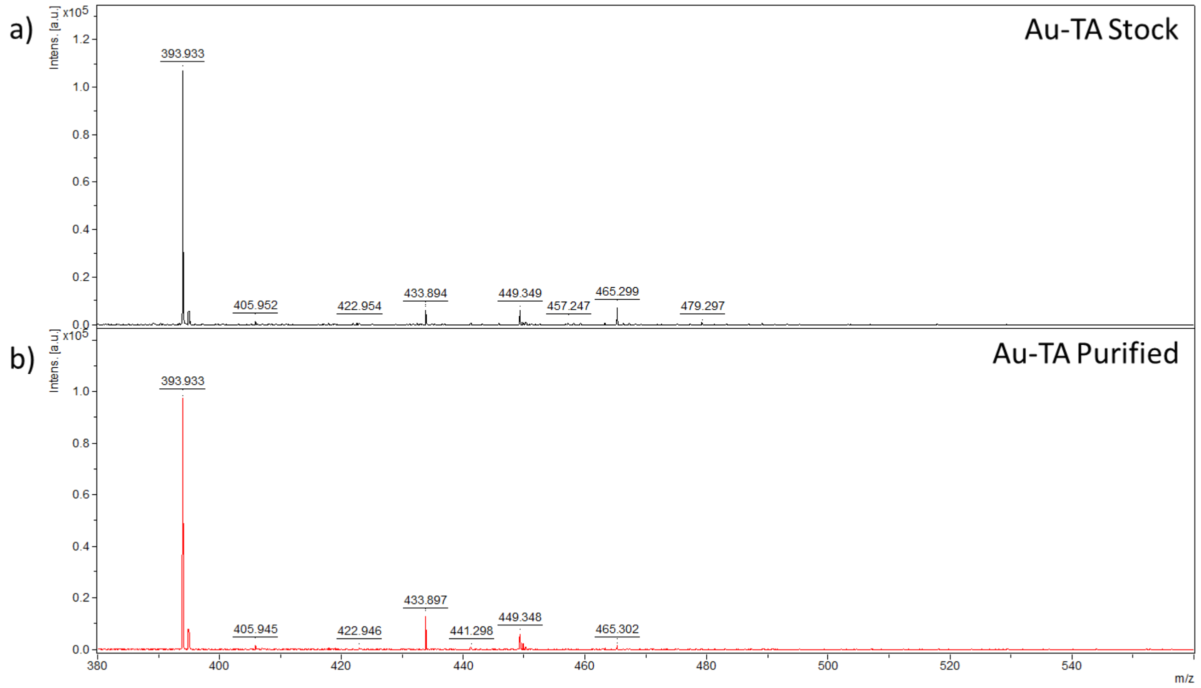

Fig. S10 Raw spectra of TA capped Au NPs in the mass range of *m/z* 380-560 in the positive mode for a) the stock and b) purified suspension

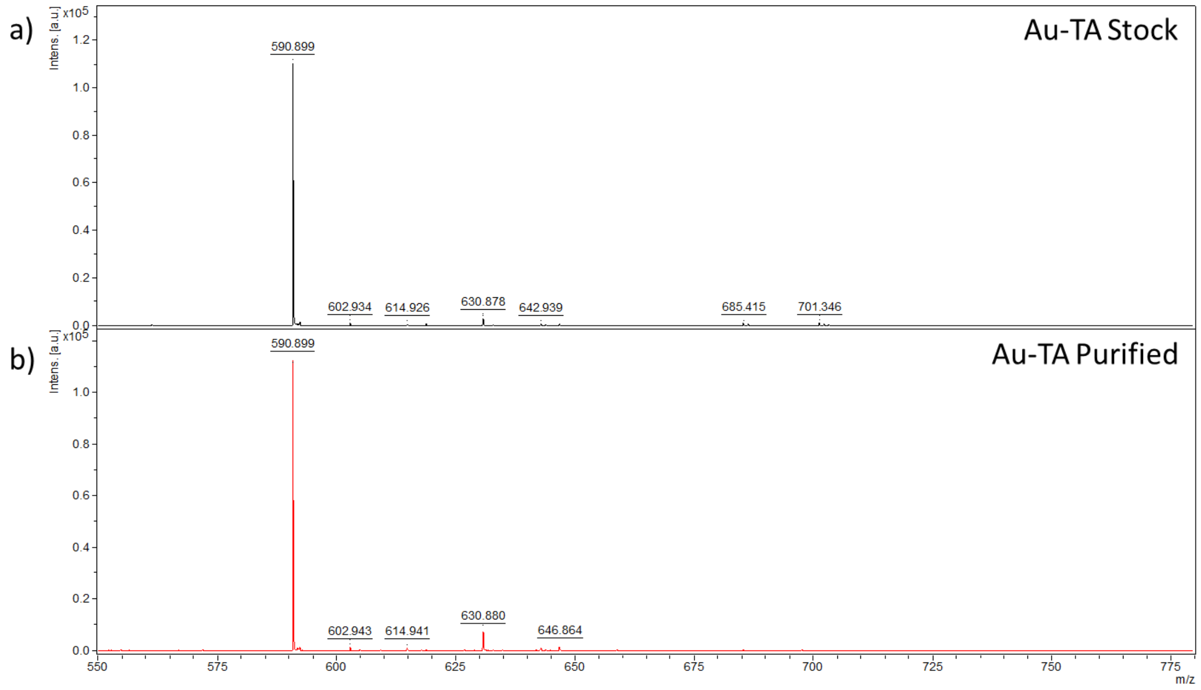

Fig. S11 Raw spectra of TA capped Au NPs in the mass range of *m/z* 550-780 in the positive mode for a) the stock and b) purified suspension

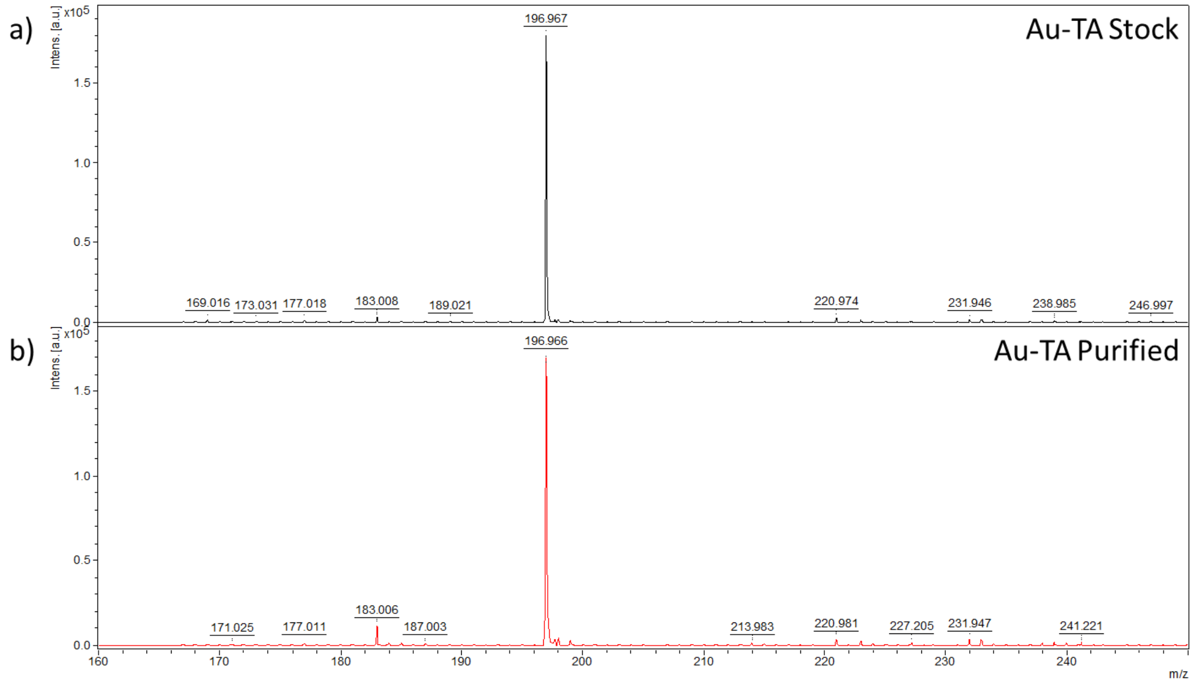

Fig. S12 Raw spectra of TA capped Au NPs in the mass range of *m/z* 160-250 in the negative mode for a) the stock and b) purified suspension. The deflection mass was set for this measurement to 200

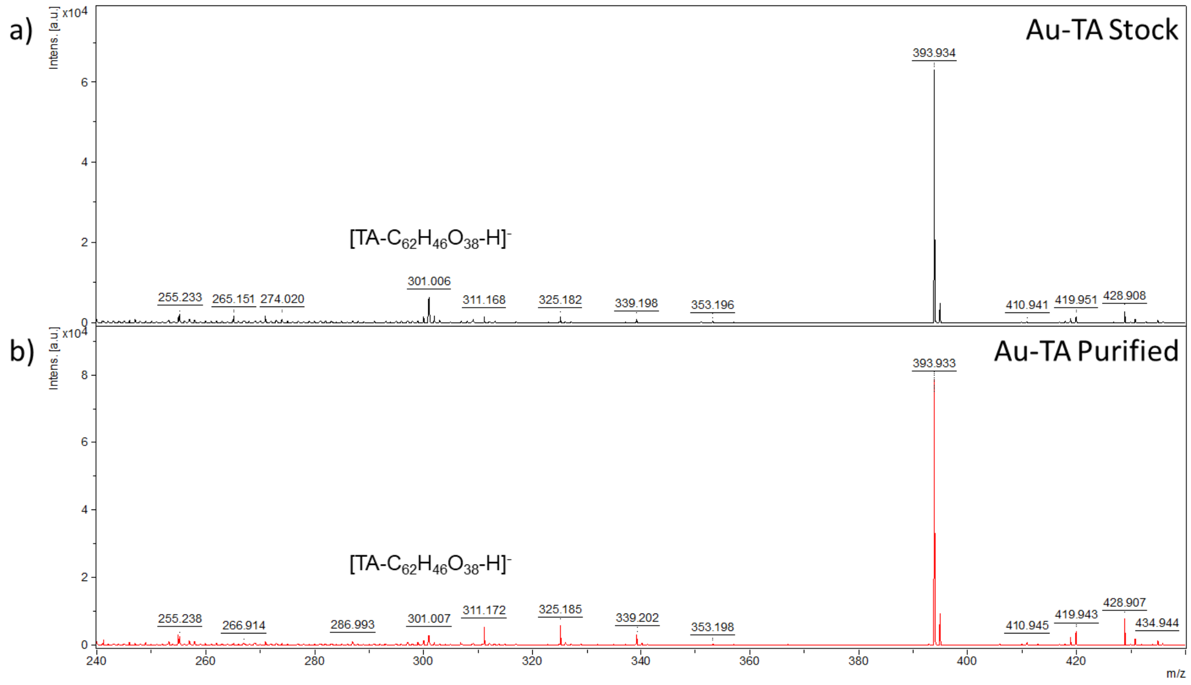

Fig. S13 Raw spectra of TA capped Au NPs in the mass range of *m/z* 240-440 in the negative mode for a) the stock and b) purified suspension. The deflection mass was set for this measurement to 200

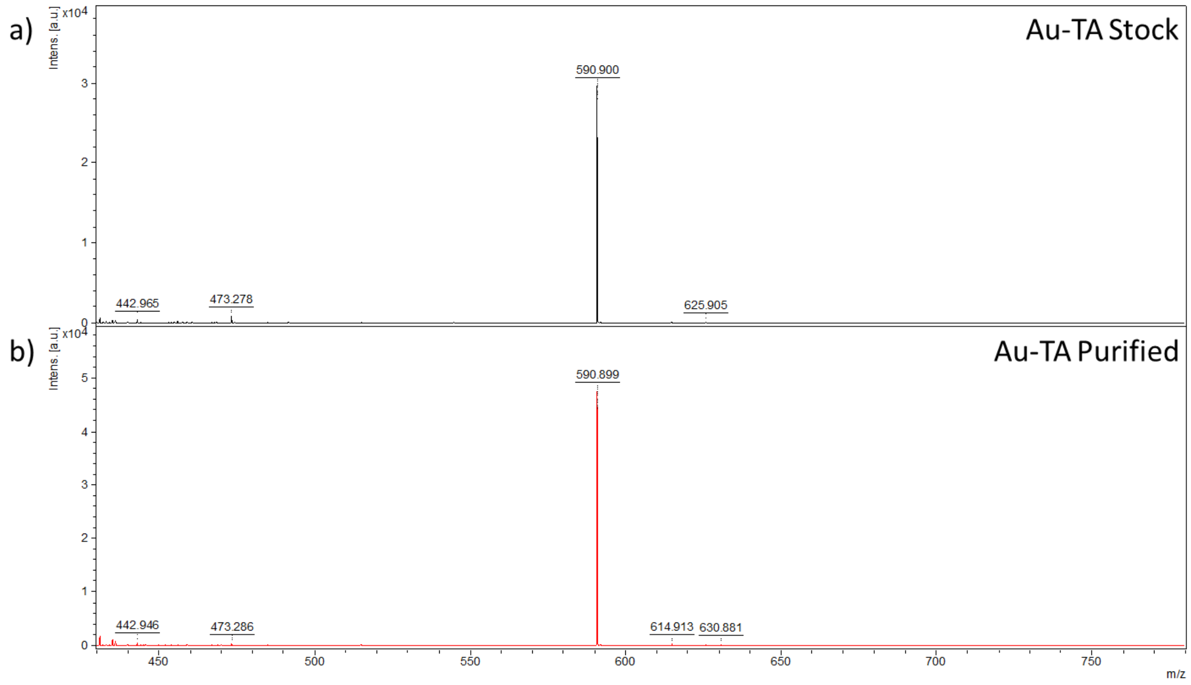

Fig. S14 Raw spectra of TA capped Au NPs in the mass range of m/z 430-780 in the negative mode for a) the stock and b) purified suspension. The deflection mass was set for this measurement to 200

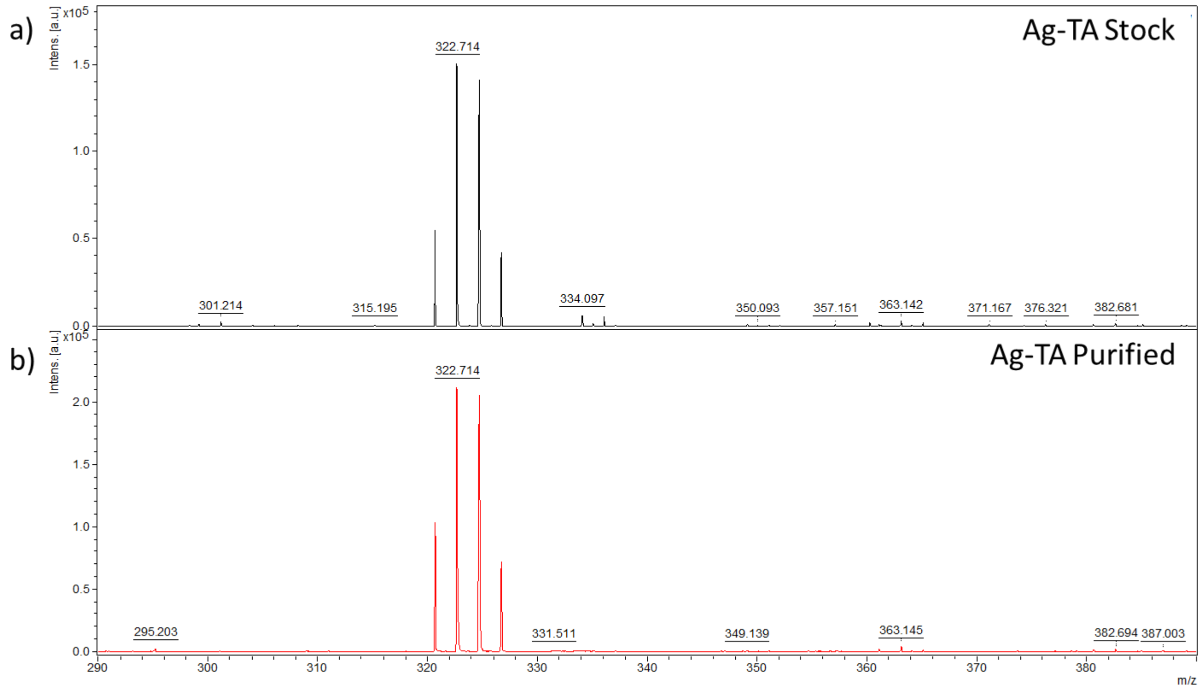

Fig. S15 Raw spectra of TA capped Ag NPs in the mass range of *m/z* 290-390 in the positive mode for a) the stock and b) purified suspension

Ag-TA from *m/z* 290-780

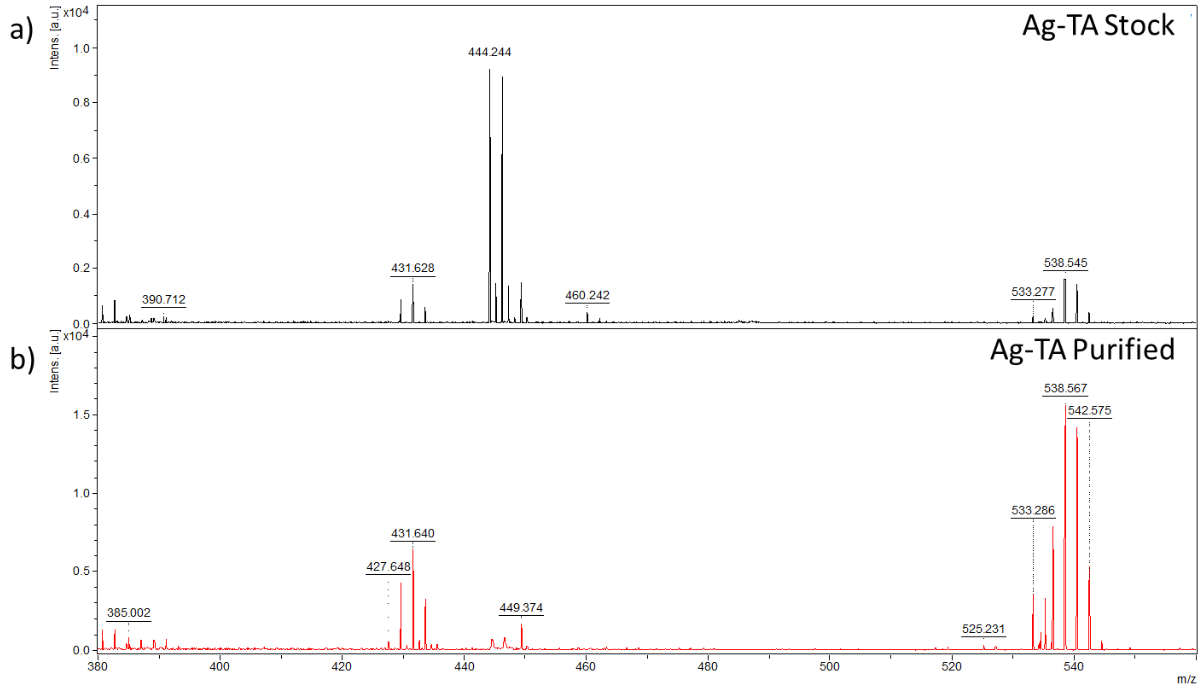

Fig. S16 Raw spectra of TA capped Ag NPs in the mass range of *m/z* 380-560 in the positive mode for a) the stock and b) purified suspension

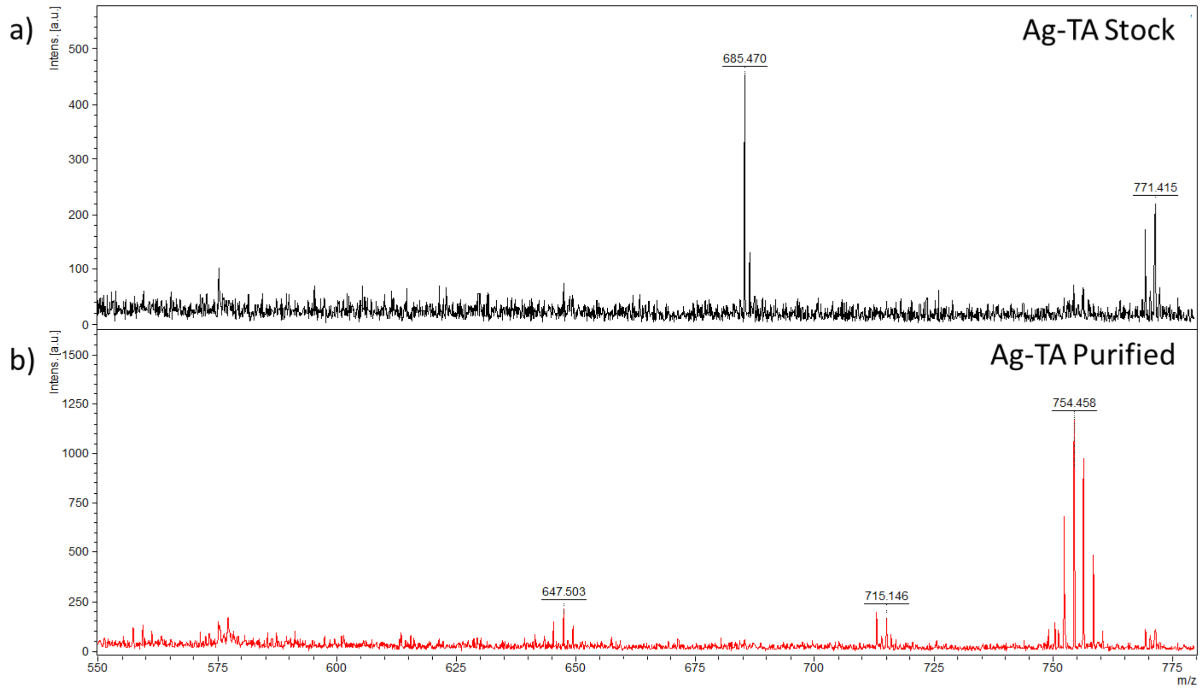

Fig. S17 Raw spectra of TA capped Ag NPs in the mass range of *m/z* 550-780 in the positive mode for a) the stock and b) purified suspension

Au-LA from *m/z* 145-780

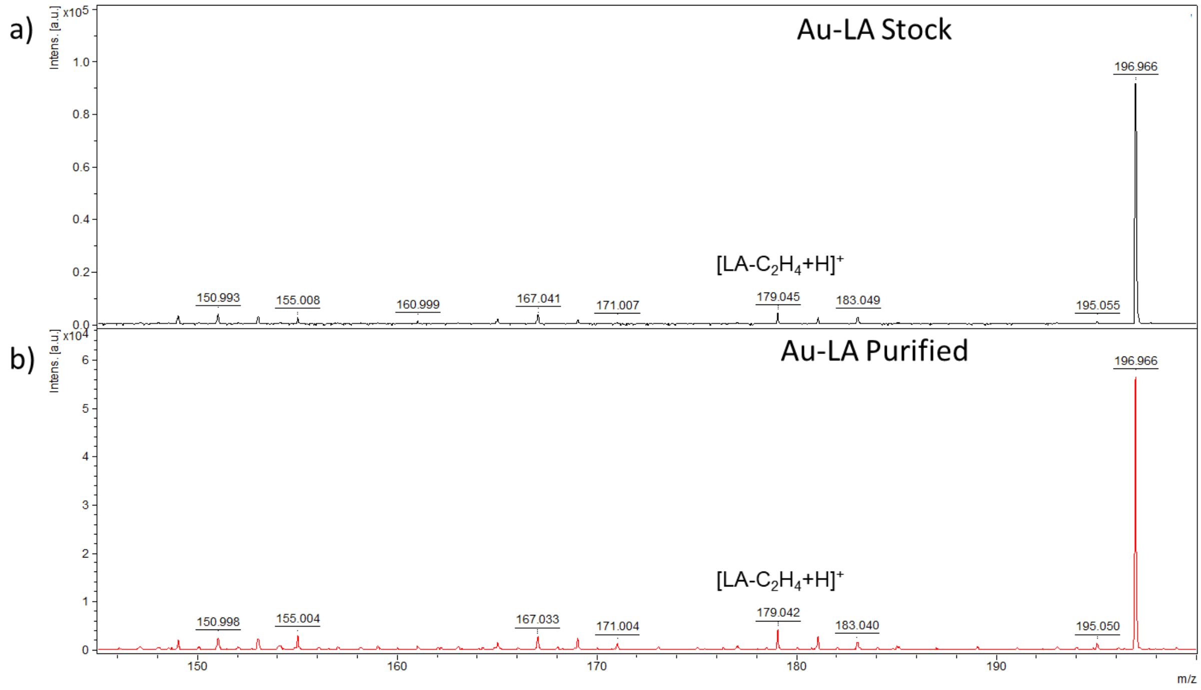

Fig. S18 Raw spectra of LA capped Au NPs in the mass range of *m/z* 145-200 in the positive mode for a) the stock and b) purified suspension

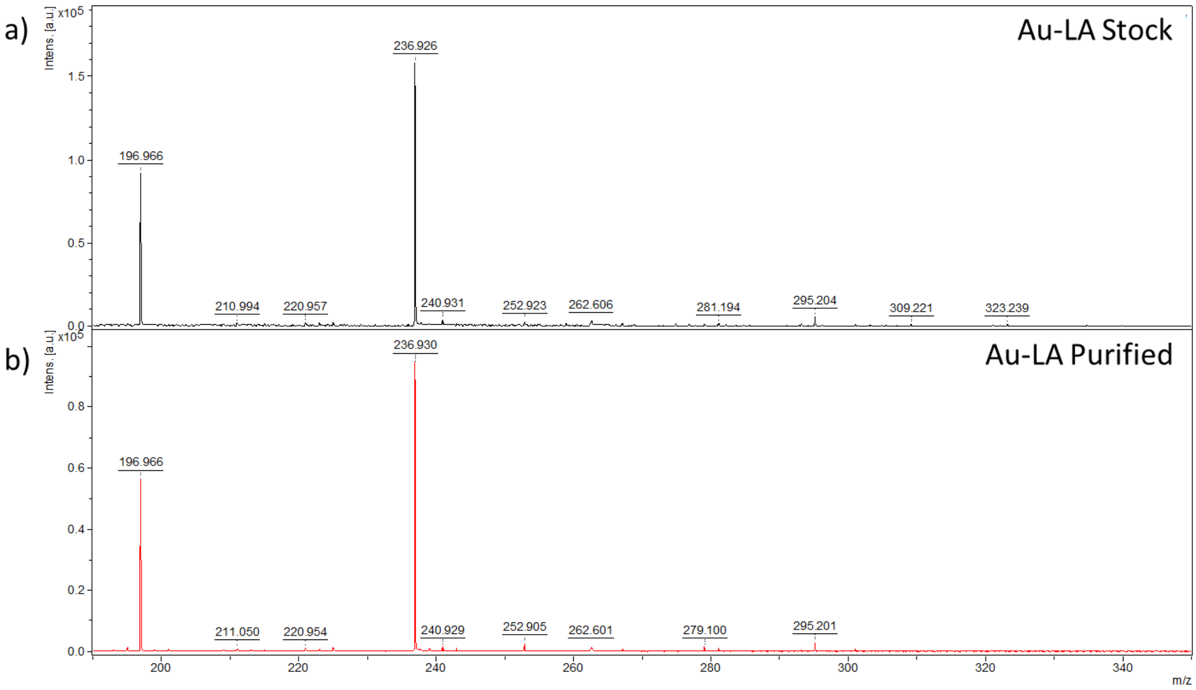

Fig. S19 Raw spectra of LA capped Au NPs in the mass range of *m/z* 190-350 in the positive mode for a) the stock and b) purified suspension

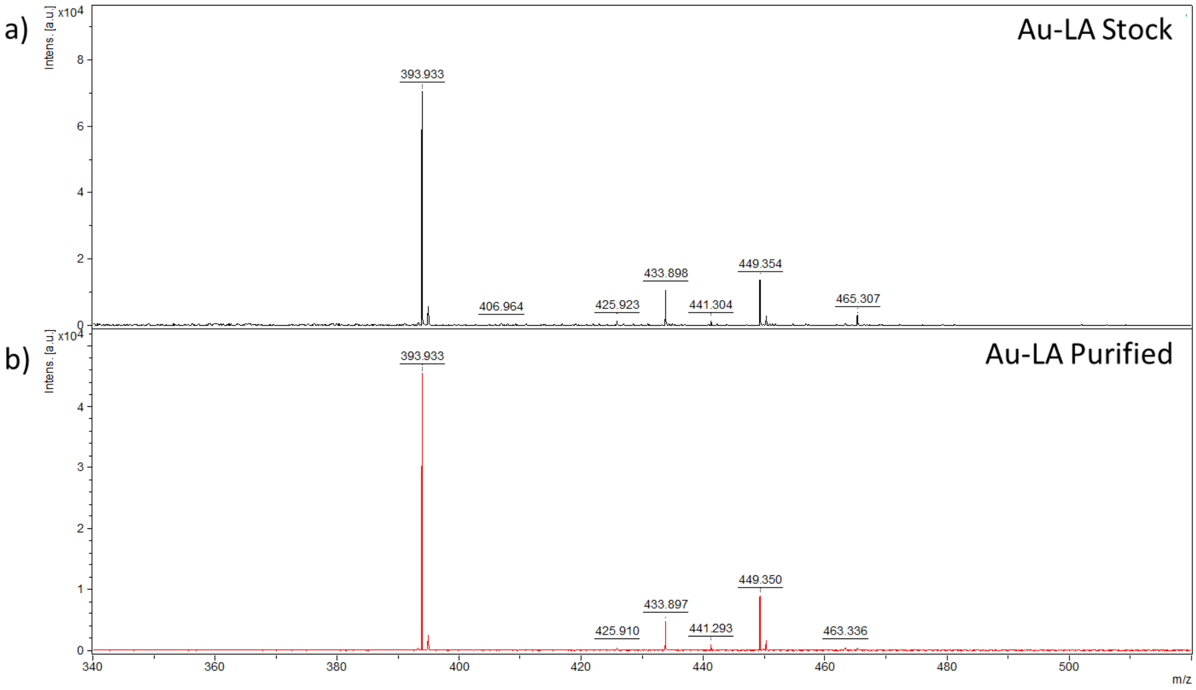

Fig. S20 Raw spectra of LA capped Au NPs in the mass range of *m/z* 340-520 in the positive mode for a) the stock and b) purified suspension

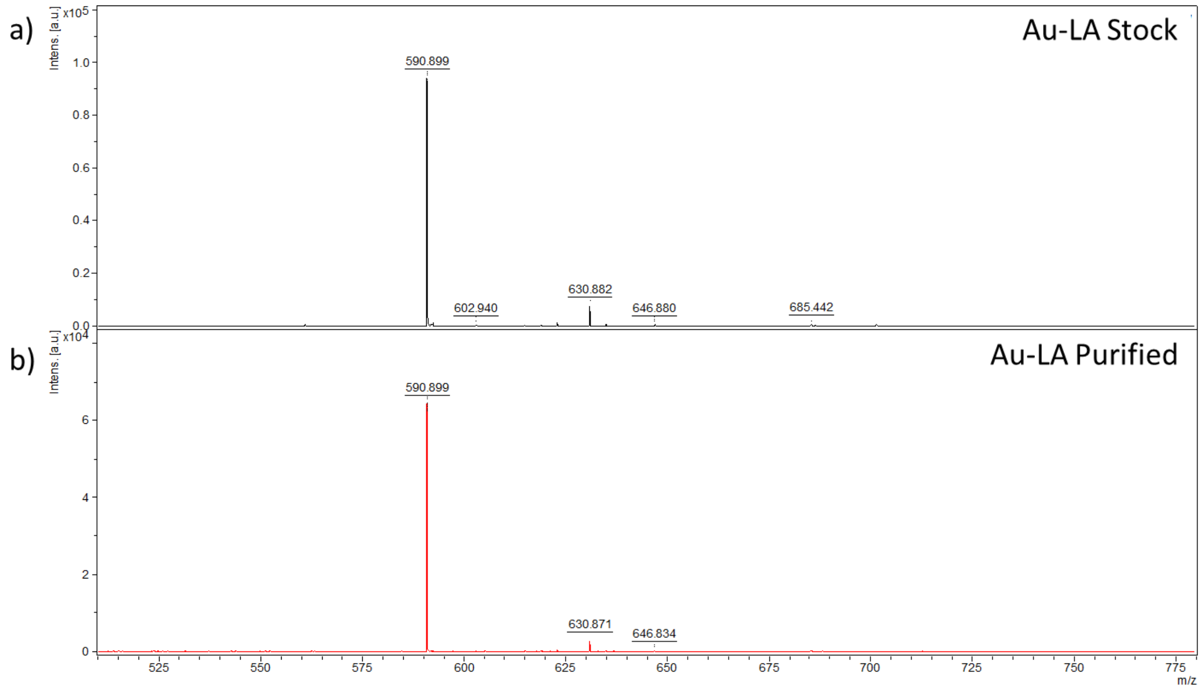

Fig. S21 Raw spectra of LA capped Au NPs in the mass range of *m/z* 510-780 in the positive mode for a) the stock and b) purified suspension

Ag-LA from *m/z* 145-780

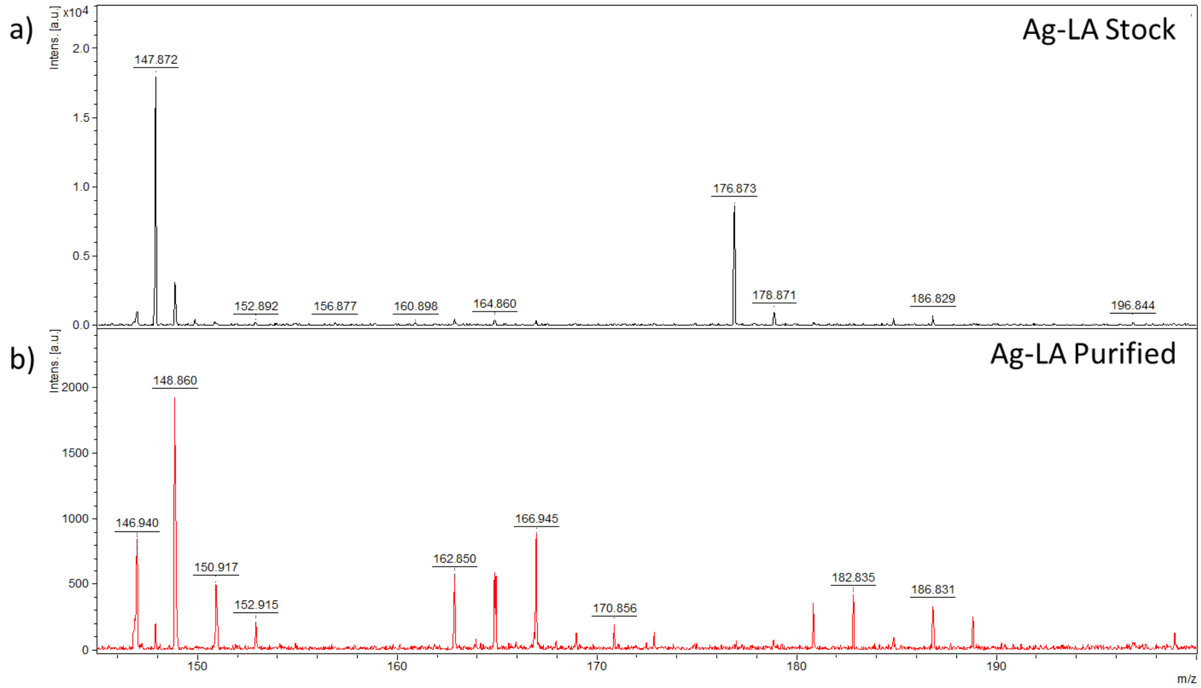

Fig. S22 Raw spectra of LA capped Ag NPs in the mass range of *m/z* 145-200 in the positive mode for a) the stock and b) purified suspension

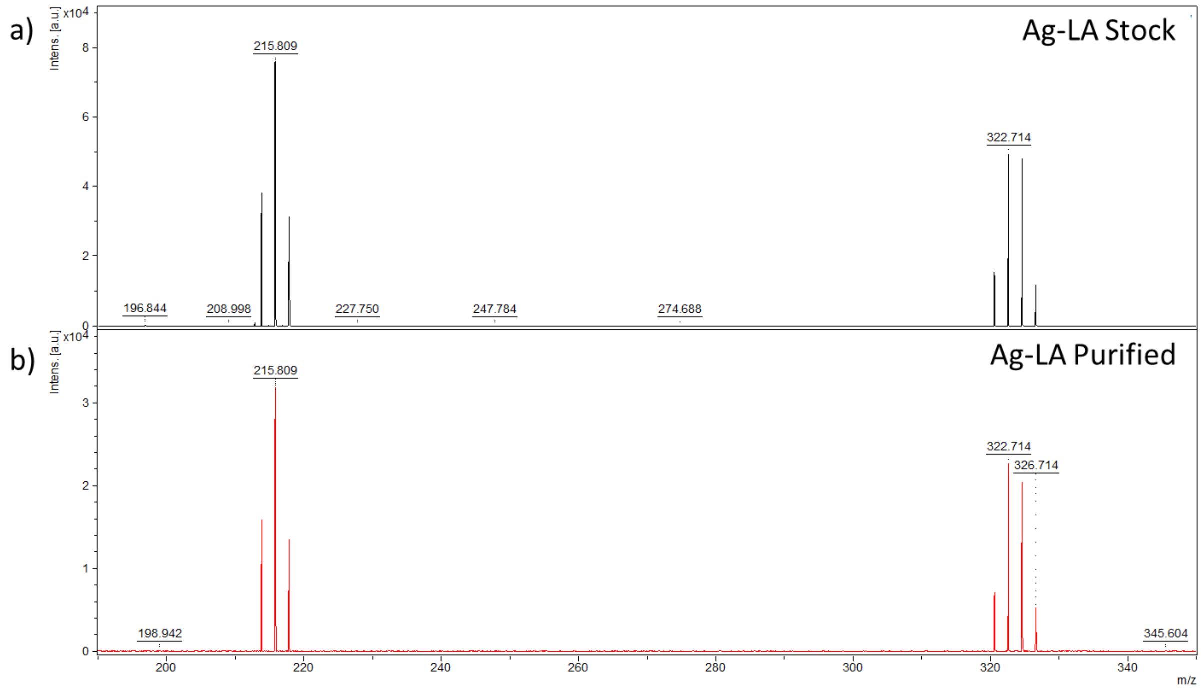

Fig. S23 Raw spectra of LA capped Ag NPs in the mass range of *m/z* 190-350 in the positive mode for a) the stock and b) purified suspension

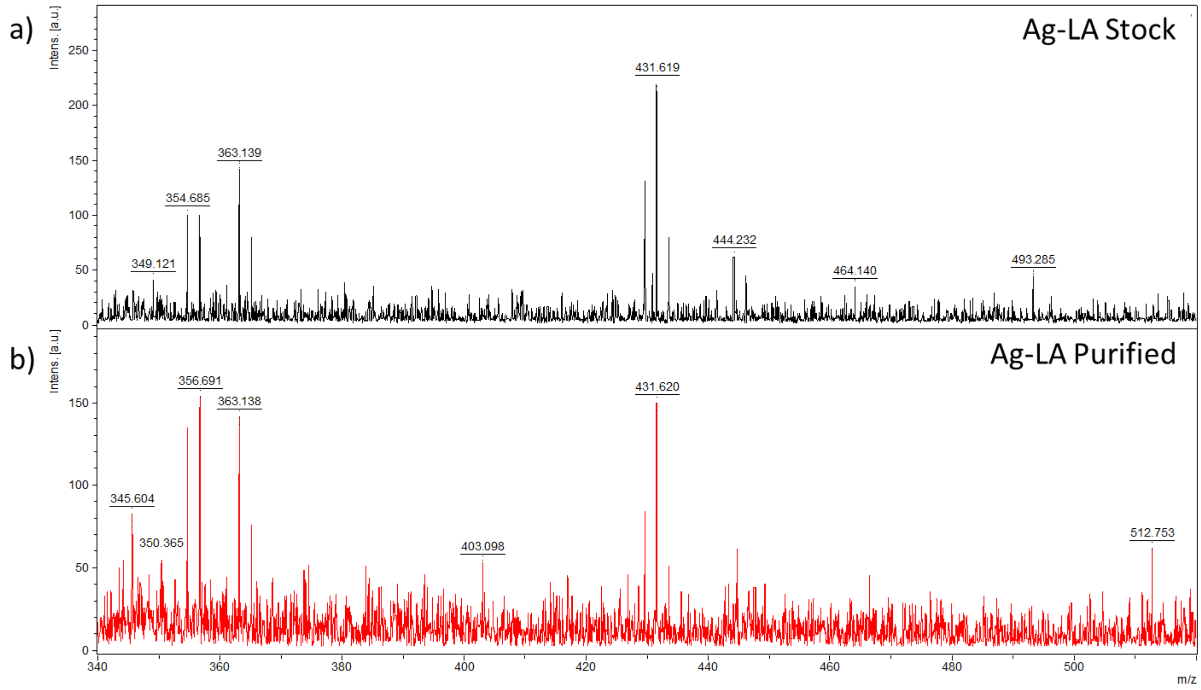

Fig. S24 Raw spectra of LA capped Ag NPs in the mass range of *m/z* 340-520 in the positive mode for a) the stock and b) purified suspension

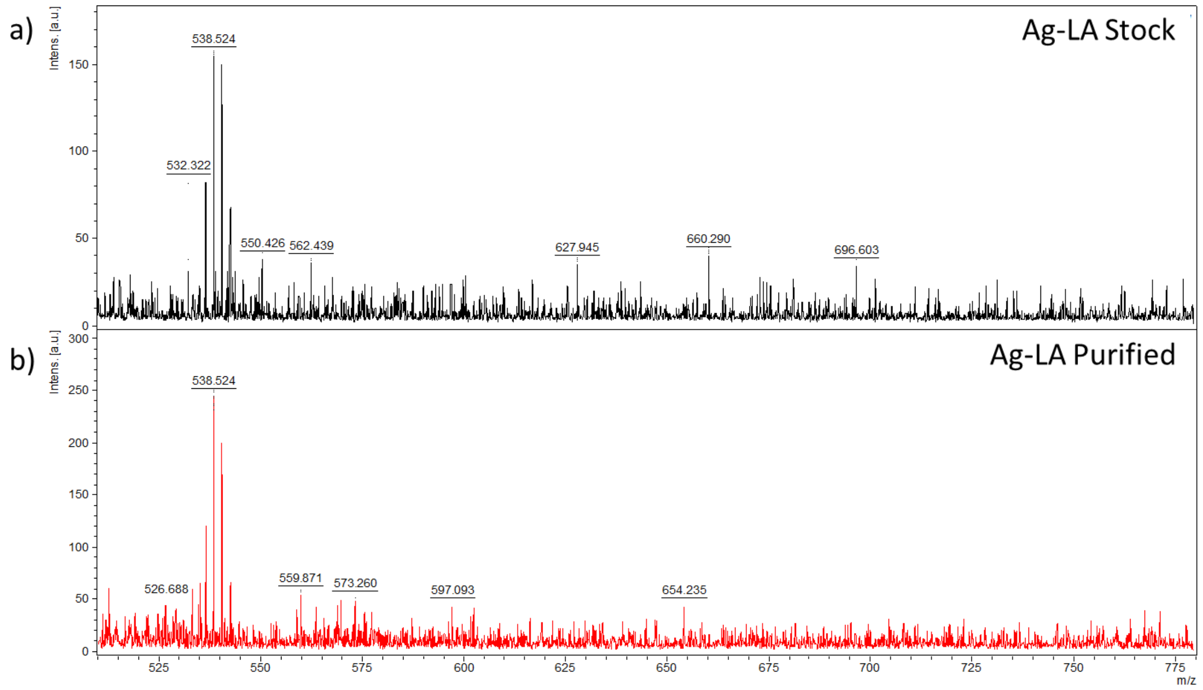

Fig. S25 Raw spectra of LA capped Ag NPs in the mass range of *m/z* 510-780 in the positive mode for a) the stock and b) purified suspension

Au-PVP from *m/z* 80-260

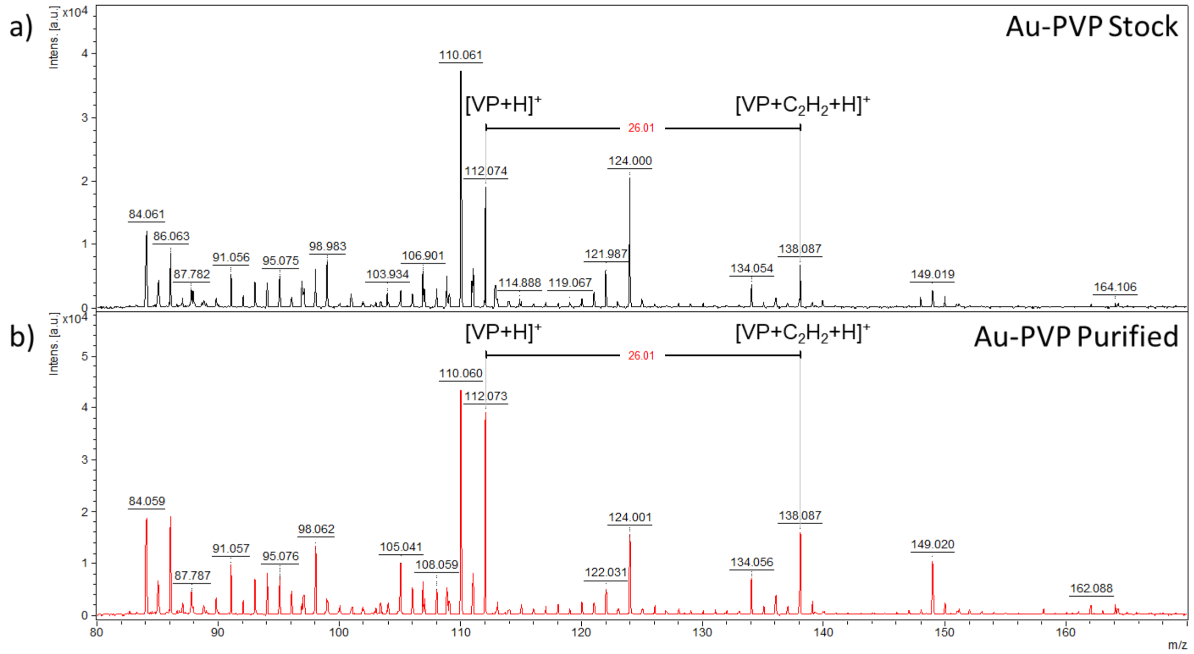

Fig. S26 Raw spectra of PVP capped Au NPs in the mass range of *m/z* 80-170 in the positive mode for a) the stock and b) purified suspension

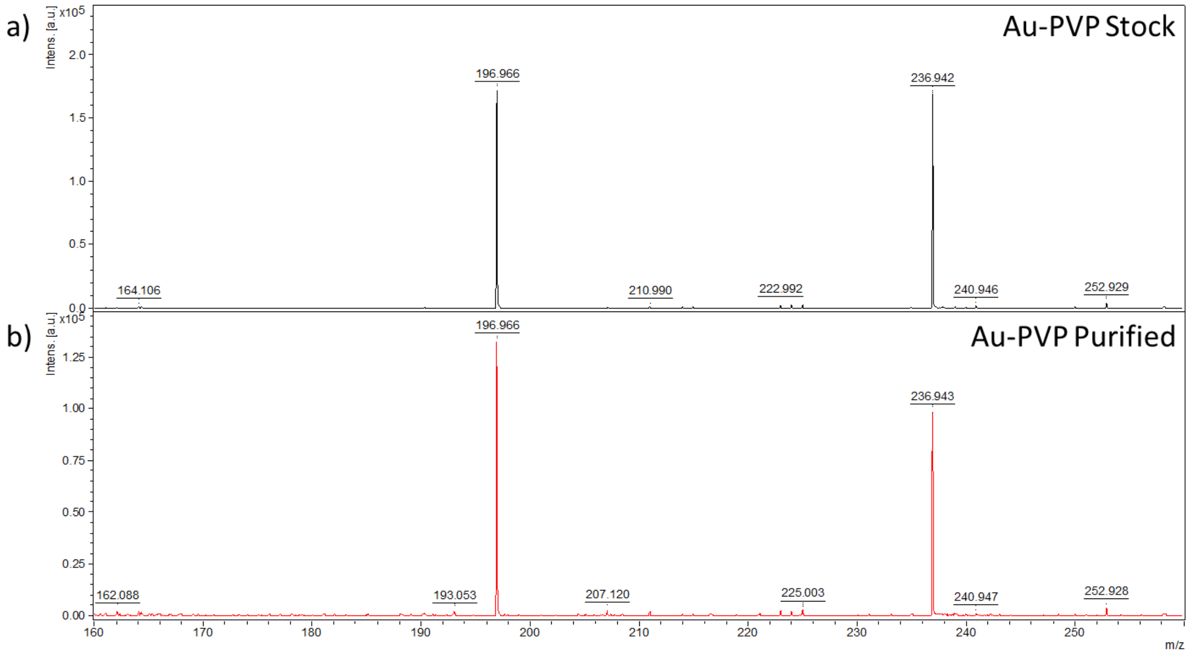

Fig. S27 Raw spectra of PVP capped Au NPs in the mass range of *m/z* 160-260 in the positive mode for a) the stock and b) purified suspension

Ag-PVP from *m/z* 80-260

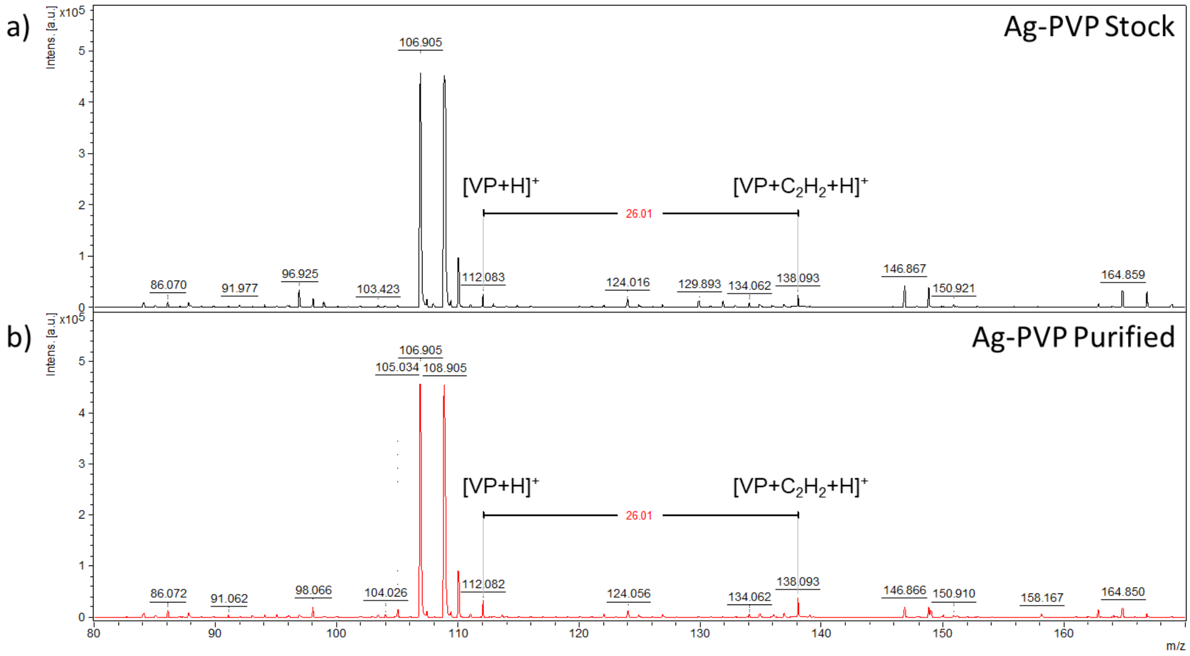

Fig. S28 Raw spectra of PVP capped Ag NPs in the mass range of *m/z* 80-170 in the positive mode for a) the stock and b) purified suspension

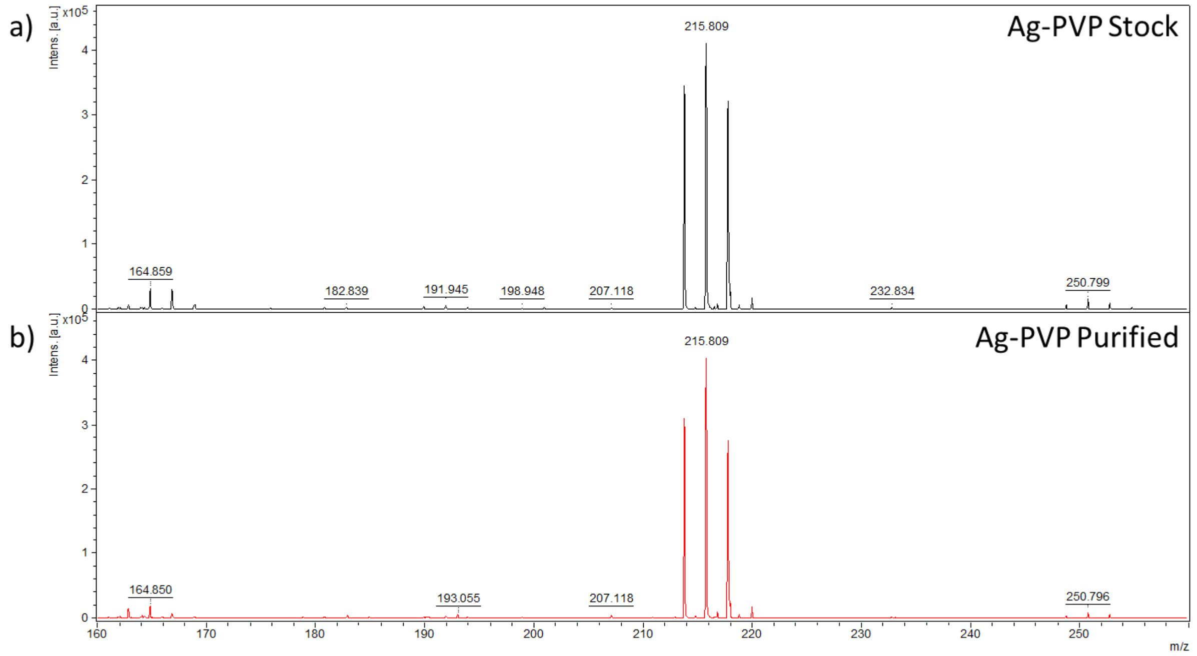

Fig. S29 Raw spectra of PVP capped Ag NPs in the mass range of *m/z* 160-260 in the positive mode for a) the stock and b) purified suspension

Au-BPEI from *m/z* 40-260

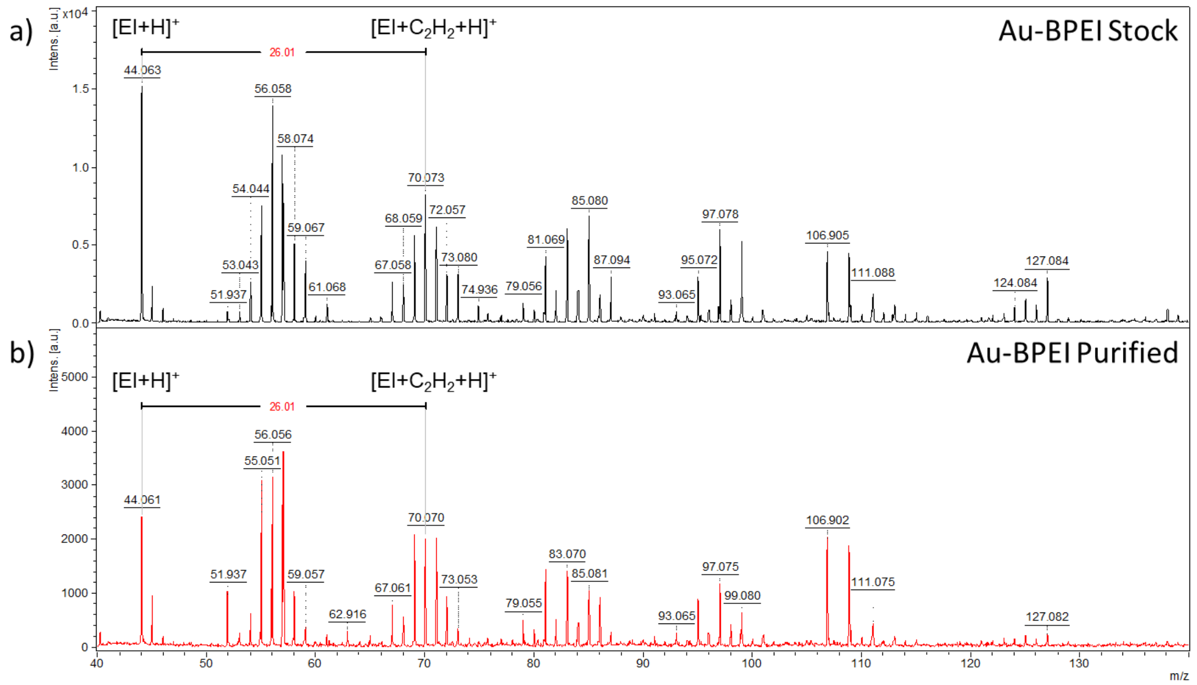

Fig. S30 Raw spectra of BPEI capped Au NPs in the mass range of *m/z* 40-140 in the positive mode for a) the stock and b) purified suspension

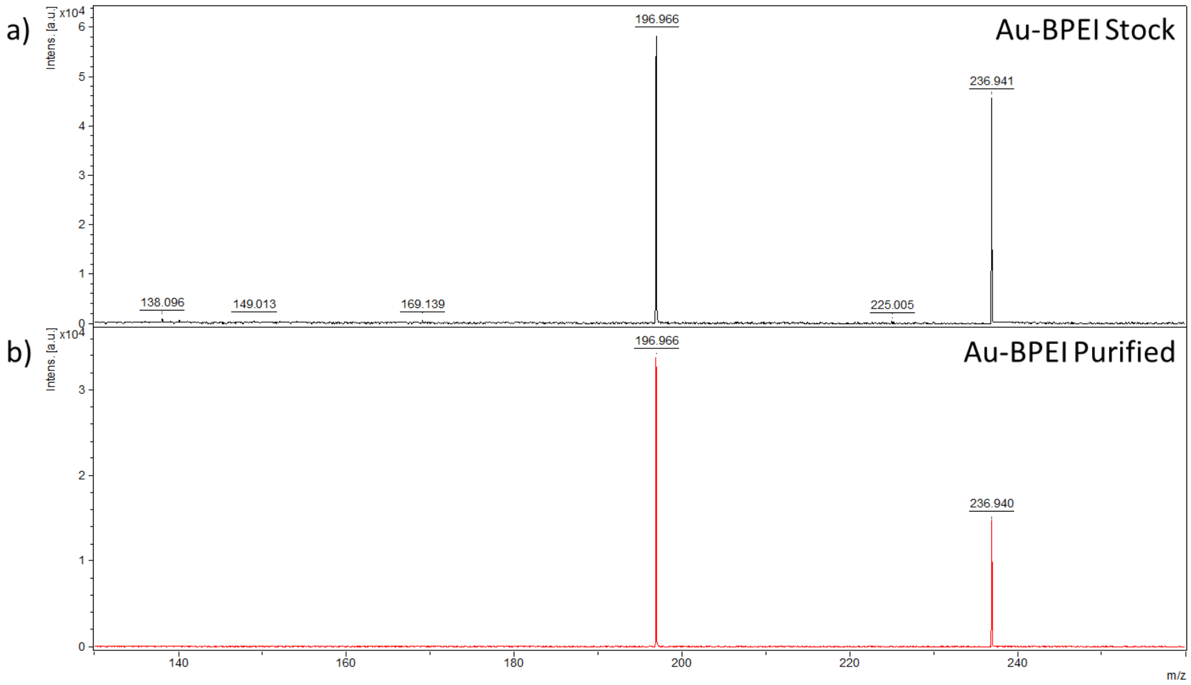

Fig. S31 Raw spectra of BPEI capped Au NPs in the mass range of *m/z* 130-260 in the positive mode for a) the stock and b) purified suspension

Ag-BPEI from *m/z* 40-260

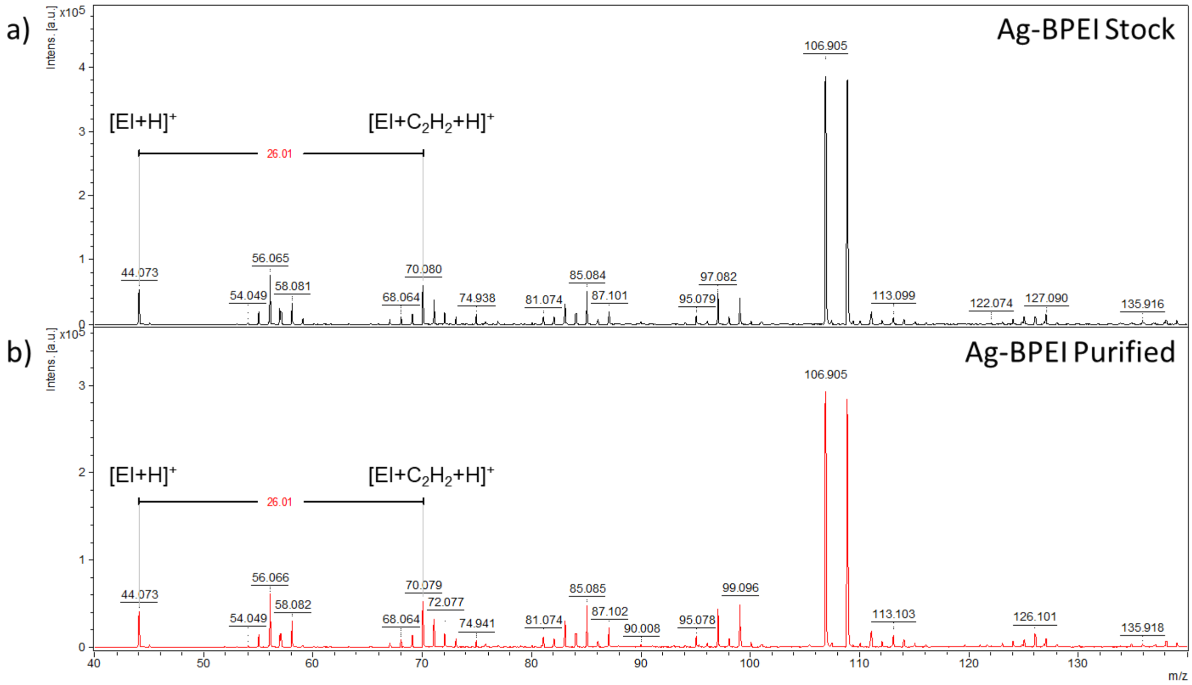

Fig. S32 Raw spectra of BPEI capped Ag NPs in the mass range of *m/z* 40-140 in the positive mode for a) the stock and b) purified suspension

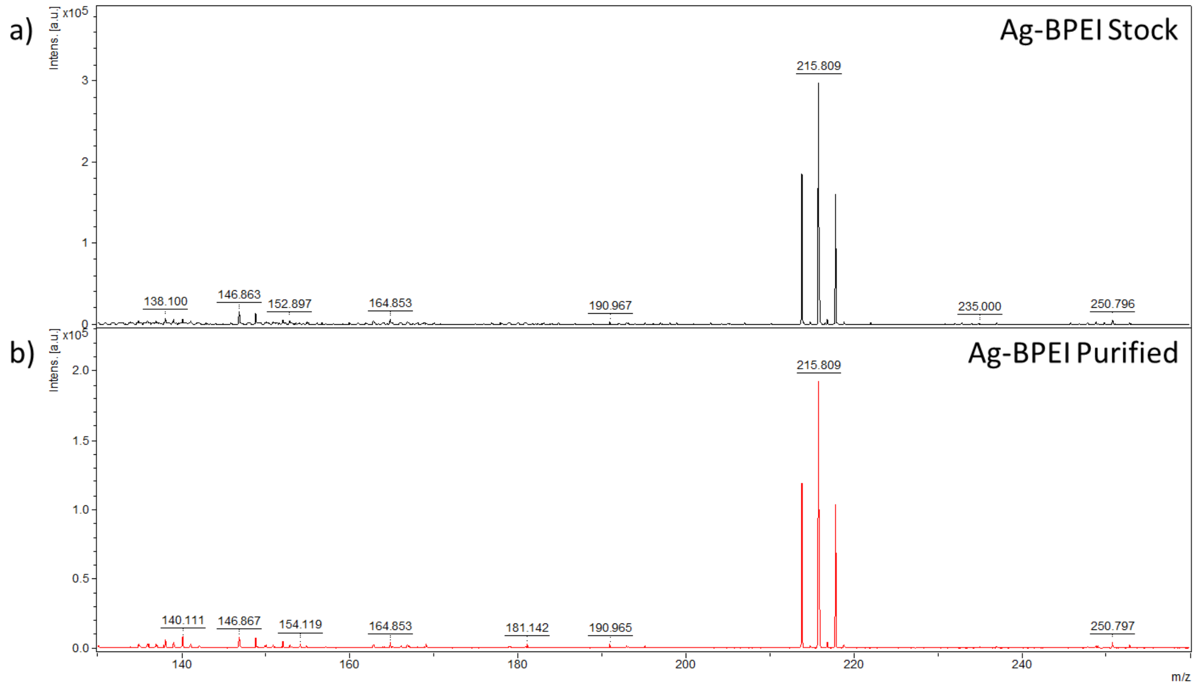

Fig. S33 Raw spectra of BPEI capped Ag NPs in the mass range of *m/z* 130-260 in the positive mode for a) the stock and b) purified suspension

Au-(m-PEG-SH) from *m/z* 40-260

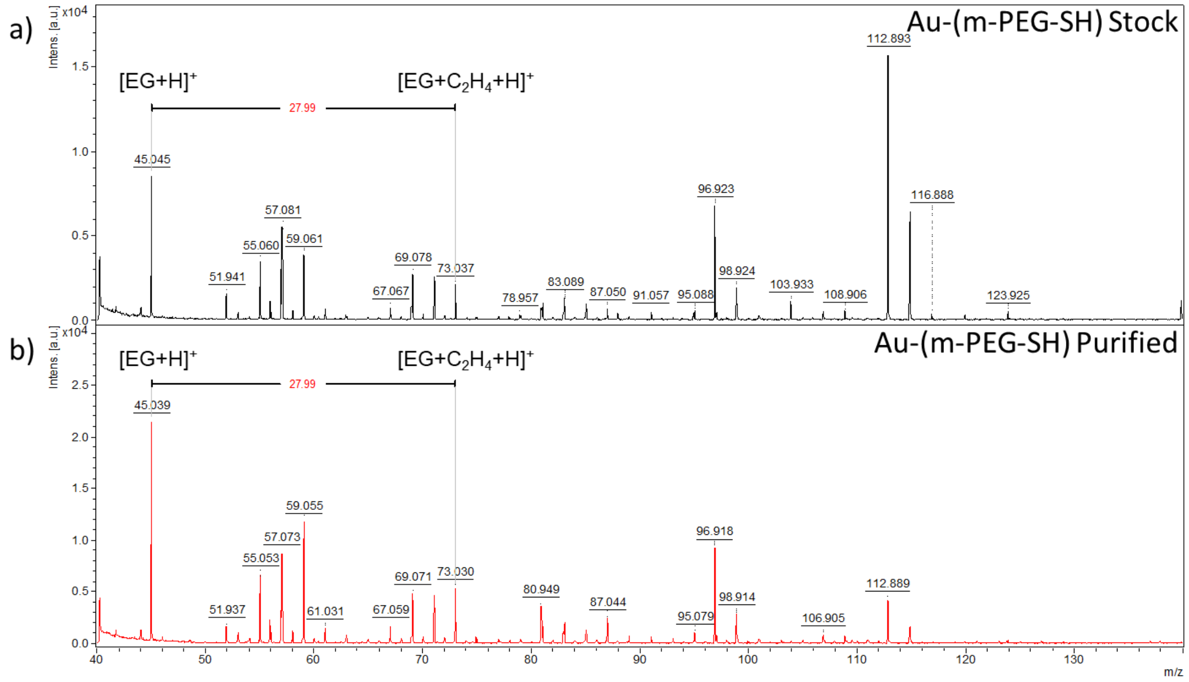

Fig. S34 Raw spectra of m-PEG-SH capped Au NPs in the mass range of *m/z* 40-140 in the positive mode for a) the stock and b) purified suspension

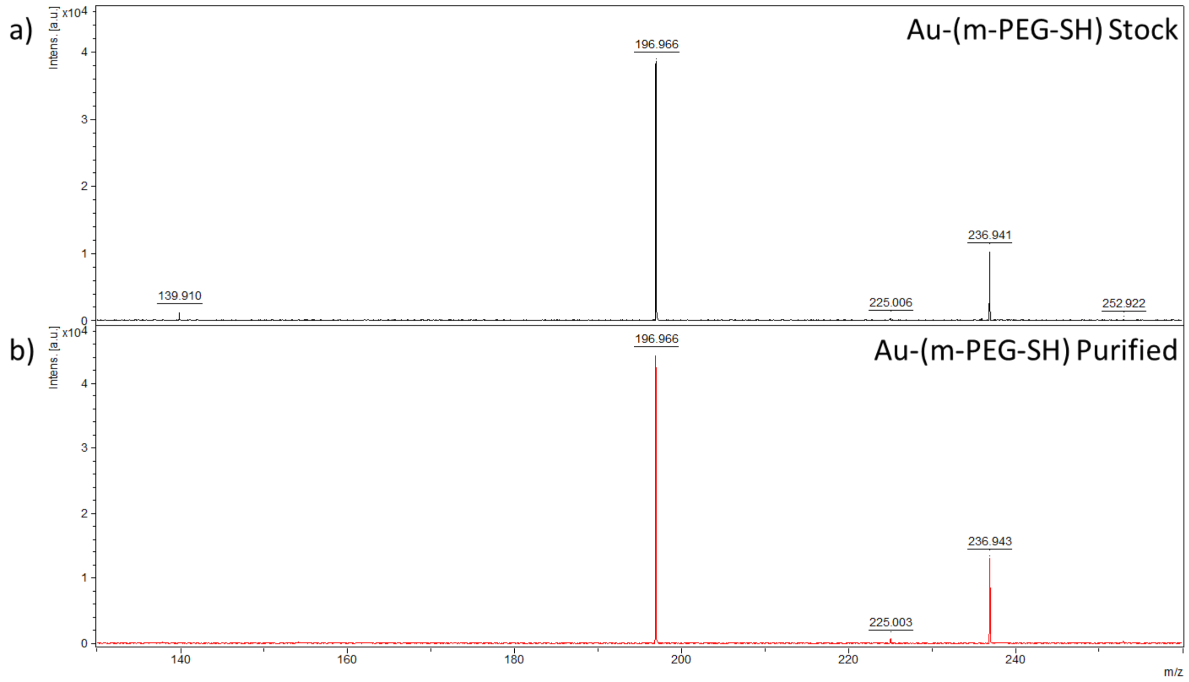

Fig. S35 Raw spectra of m-PEG-SH capped Au NPs in the mass range of *m/z* 130-260 in the positive mode for a) the stock and b) purified suspension

Ag-(m-PEG-SH) from *m/z* 40-260

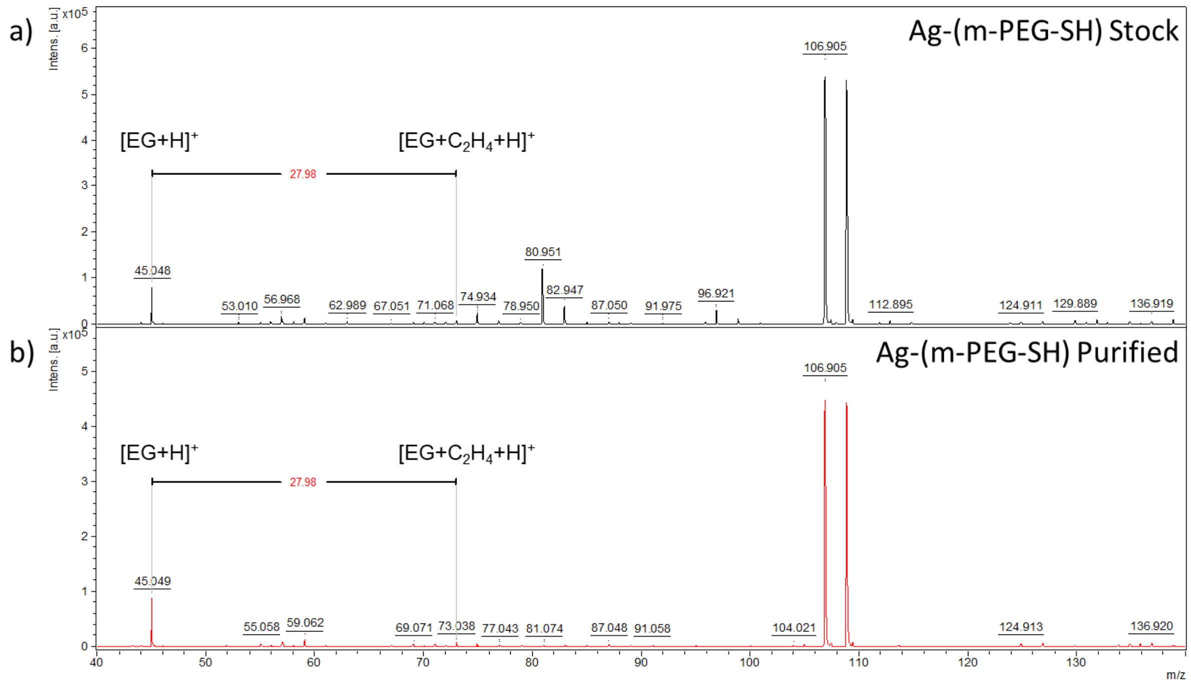

Fig. S36 Raw spectra of m-PEG-SH capped Ag NPs in the mass range of *m/z* 40-140 in the positive mode for a) the stock and b) purified suspension

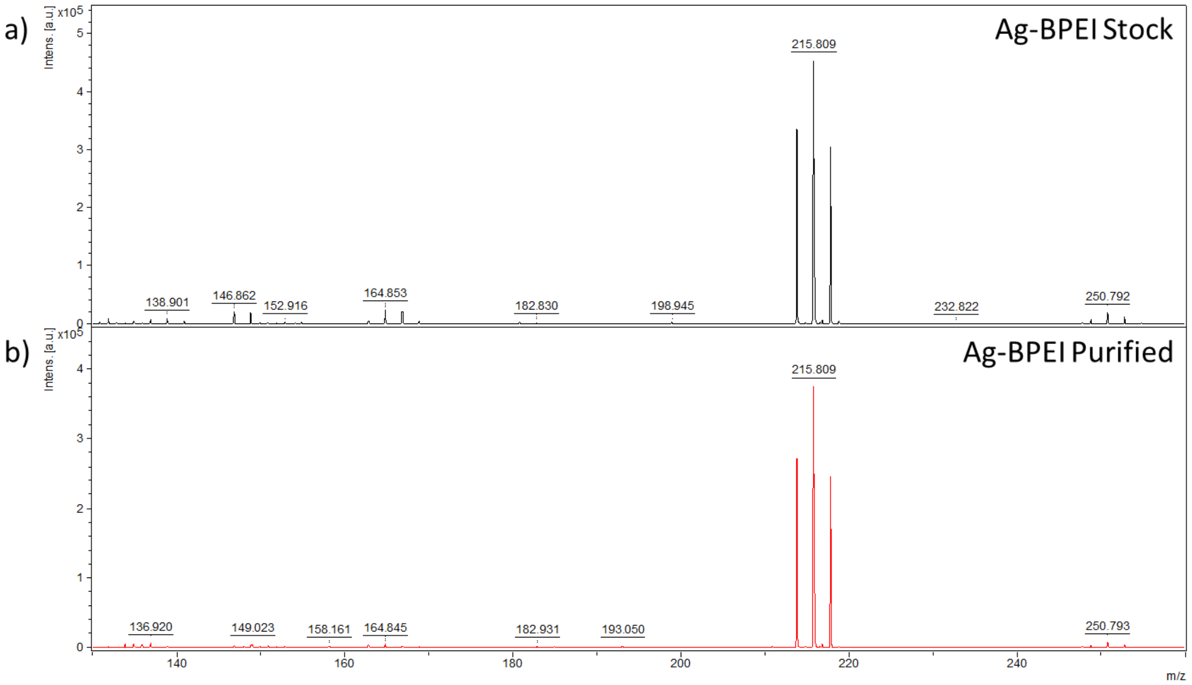

Fig. S37 Raw spectra of m-PEG-SH capped Ag NPs in the mass range of *m/z* 130-260 in the positive mode for a) the stock and b) purified suspension

Table S1 Summary of all identified characteristic capping agent signals together with the errors for Au and Ag NPs of the stock and purified suspensions

Capping agent	Molecular formula	Identity	Exact mass [m/z]	Au NPs						Ag NPs					
				Stock			Purified			Stock			Purified		
				Accurate mass [m/z]	Δm [mDa]	Δm [ppm]	Accurate mass [m/z]	Δm [mDa]	Δm [ppm]	Accurate mass [m/z]	Δm [mDa]	Δm [ppm]	Accurate mass [m/z]	Δm [mDa]	Δm [ppm]
Citric acid (CA)	$C_6H_8O_7$	$[CA+H]^+$	193.034279	192.977	-57	-297	193.006	-28	-146	---	---	---	193.059	25	128
Tannic acid (TA)	$C_{76}H_{52}O_{46}$	$[TA-C_{62}H_{46}O_{38}+H]^+$	303.013544	303.040	26	87	303.026	12	41	---	---	---	---	---	---
		$[TA-C_{62}H_{46}O_{38}-H]^-$	300.998991	301.006	7	23	301.007	8	27	---	---	---	---	---	---
Lipoic acid (LA)	$C_8H_{14}O_2S_2$	$[LA-C_2H_4+H]^+$	179.019498	179.045	26	142	179.042	23	126	---	---	---	---	---	---
Polyvinyl-pyrrolidone (PVP)	C_6H_9NO	$[VP+H]^+$	112.075690	112.074	-2	-15	112.073	-3	-24	112.083	7	65	112.082	6	56
		$[VP+C_2H_2+H]^+$	138.091340	138.087	-4	-31	138.087	-4	-31	138.093	2	12	138.093	2	12
Branched polyethylen-imine (BPEI)	C_2H_5N	$[EI+H]^+$	44.049476	44.063	14	307	44.061	12	262	44.073	24	534	44.073	24	534
		$[EI+C_2H_2+H]^+$	70.065126	70.073	8	112	70.070	5	70	70.080	15	212	70.079	14	198
Polyethylene glycol (PEG)	C_2H_4O	$[EG+H]^+$	45.033491	45.045	12	256	45.039	6	122	45.048	15	322	45.049	16	344
		$[EG+C_2H_4+H]^+$	73.064791	73.037	-28	-380	73.030	-35	-476	73.038	-27	-367	73.038	-27	-367

"---" denotes not assigned signals due to S/N < 5

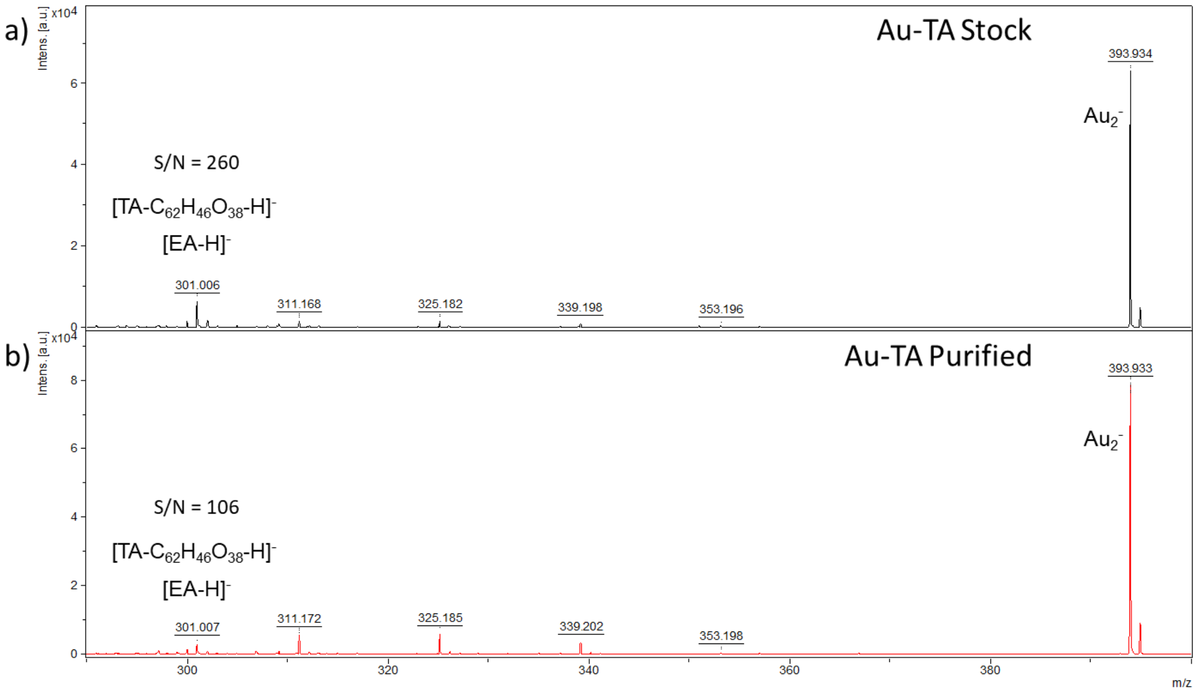

Fig. S38 Spectra of TA capped Au NPs showing the identified fragment ion of TA in the mass range of *m/z* 290-400 in the negative mode for a) the stock and b) purified suspension. The deflection mass was set for this measurement to 200

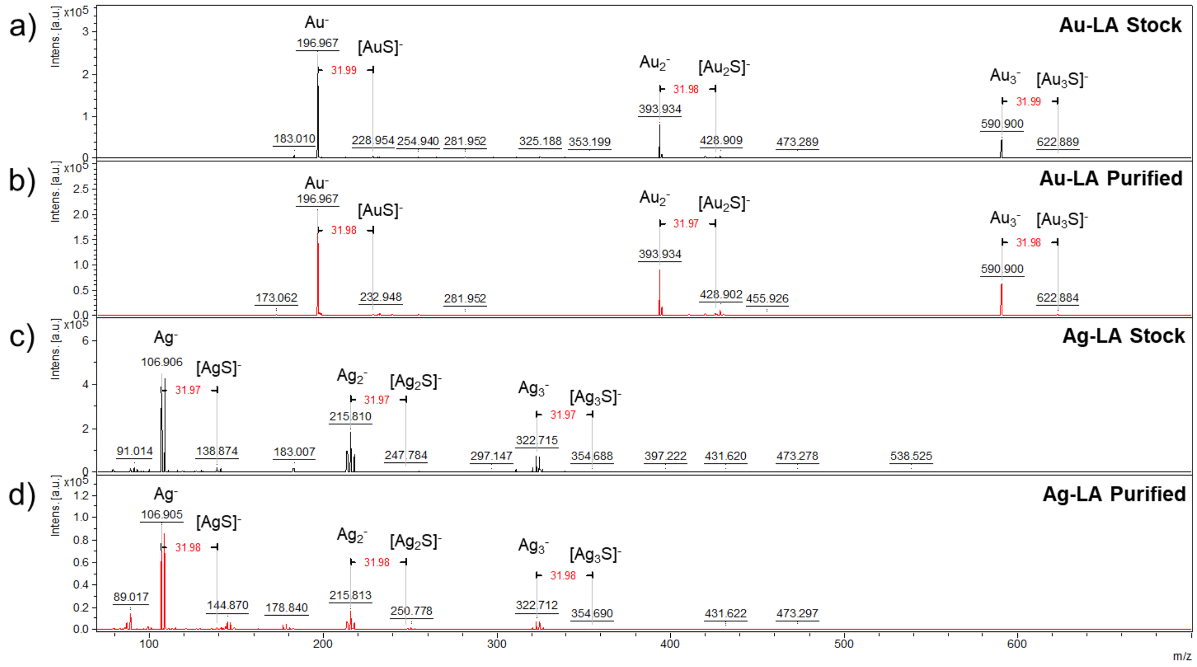

Fig. S39 Spectra of LA capped Au and Ag NPs in the negative mode for the stock and purified suspensions showing the metal cluster ions with the corresponding metal-sulfur cluster ions with the distance of a sulfur ion which has a theoretical monoisotopic neutral mass of 31.972 Da. In a) stock Au-LA, b) purified Au-LA, c) stock Ag-LA and d) purified Ag-LA

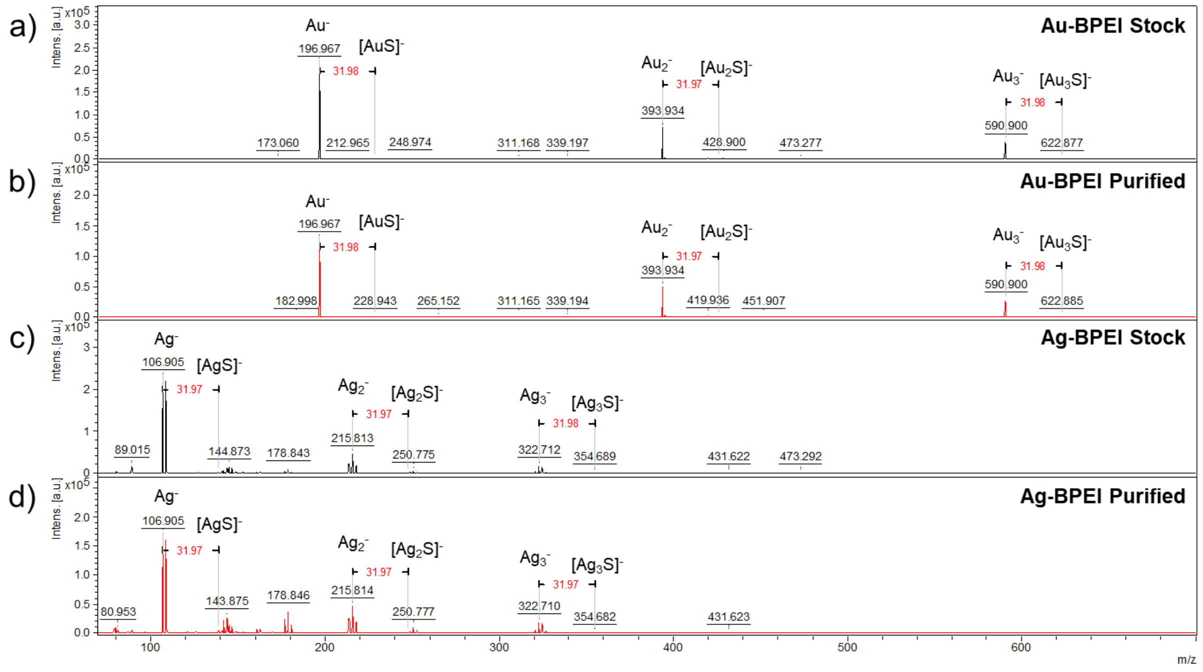

Fig. S40 Spectra of BPEI capped Au and Ag NPs in the negative mode for the stock and purified suspensions showing the metal cluster ions with the corresponding metal-sulfur cluster ions with the distance of a sulfur ion which has a theoretical monoisotopic neutral mass of 31.972 Da. In a) stock Au-BPEI, b) purified Au-BPEI, c) stock Ag-BPEI and d) purified Ag-BPEI

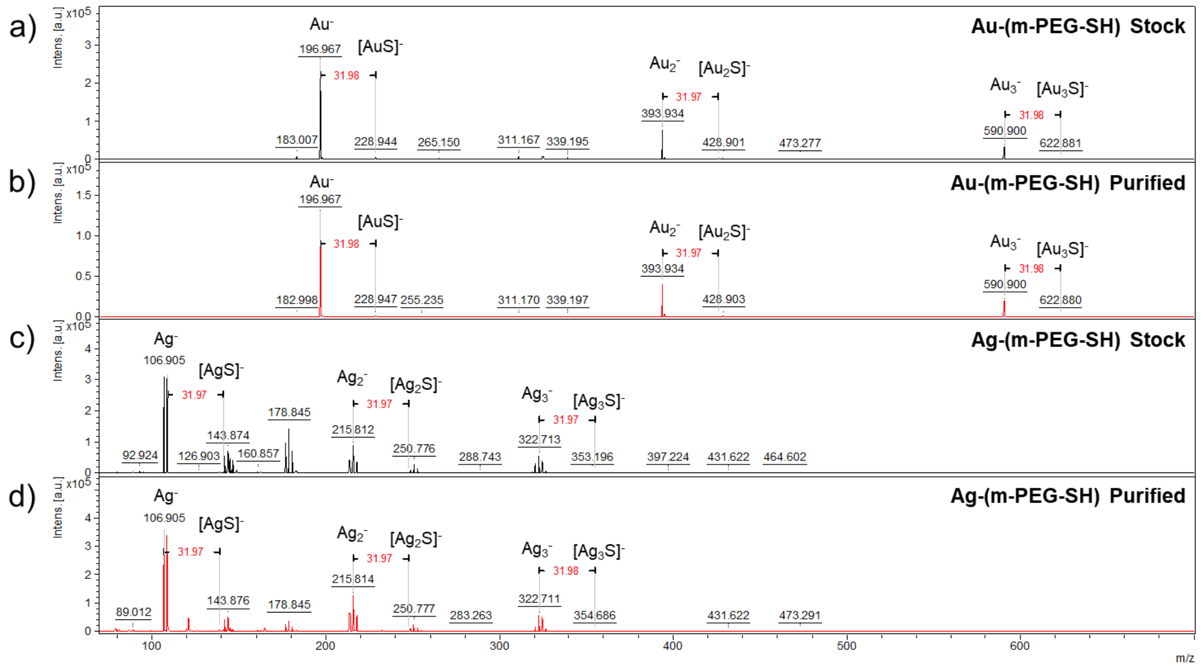

Fig. S41 Spectra of m-PEG-SH capped Au and Ag NPs in the negative mode for the stock and purified suspensions showing the metal cluster ions with the corresponding metal-sulfur cluster ions with the distance of a sulfur ion which has a theoretical monoisotopic neutral mass of 31.972 Da. In a) stock Au-(m-PEG-SH), b) purified Au-(m-PEG-SH), c) stock Ag-(m-PEG-SH) and d) purified Ag-(m-PEG-SH)

2 LDI-ToF-MS analyses of purified suspensions

Total organic carbon (TOC) analyses were used to control the progress of purification. A single centrifugation and resuspension cycle was sufficient for removing the dissolved organics and decreasing the TOC concentration of the NP suspensions to below 1 mg/L (Table S2). Because TA capped Au NPs did not show a clear purification after one centrifugation step this experiment was performed again but with two centrifugation steps where TOC was measured for both supernatants. The first supernatant yielded 2.64 ± 0.22 mg/L and the second supernatant 0.28 ± 0.05 mg/L. The measurements revealed that, two samples required two purification steps: (i) CA capped Ag NPs because the NPs are not dispersed in water but in 2 mM sodium citrate solution and (ii) TA capped Au NPs which may form stronger and specific polyphenol multi-layer structures on the Au NP surface.

Table S2 Results from the total organic carbon (TOC) measurements of the stock suspensions, the supernatants after centrifugation and the pellet which was diluted to the initial stock concentration by the addition of 2 mL MilliQ water which is referred as “purified suspension”. The results were determined by three samples (*n*=3) where each was measured trice

**NP-capping agent combination**	**TOC of stock suspension**	**TOC of supernatant**	**TOC of purified suspension**
	mean ± stdev [mg/L]	mean ± stdev [mg/L]	mean ± stdev [mg/L]
Au-PVP	11.9 ± 0.11	11.4 ± 0.18	0.15 ± 0.22
Ag-PVP	7.02 ± 0.25	6.91 ± 0.07	< LOD
Au-(m-PEG-SH)	1.28 ± 0.04	1.18 ± 0.13	< LOD
Ag-(m-PEG-SH)	4.68 ± 0.26	4.21 ± 0.21	< LOD
Au-BPEI	3.46 ± 0.44	3.61 ± 0.11	< LOD
Ag-BPEI	4.37 ± 0.42	4.54 ± 0.20	< LOD
Au-LA	1.41 ± 0.03	1.28 ± 0.09	< LOD
Ag-LA	1.68 ± 0.12	1.54 ± 0.14	< LOD
Au-CA	9.06 ± 0.24	8.78 ± 0.14	< LOD
Ag-CA	213 ± 2.22	215 ± 2.90	0.70 ± 0.23
Au-TA	4.17 ± 0.13	2.29 ± 0.15	0.13 ± 0.08
Ag-TA	6.31 ± 0.18	6.15 ± 0.06	< LOD

3 Concentration dependency of the signal intensity

Table S3 Reported molecules per nm^2 values (for small molecules termed as "ligand densities" whereas for polymers as "grafting densities") for the used capping agents reported for Au NPs

Capping agent	Molecules per nm^2	Monomers of polymers per nm^2	NP size [nm]	Molecular weight of polymer [kDa]	Methods
CA	3.1	---	~20	---	elemental analysis [1]
TA	0.2	---	---	---	approximated as a circle with an area of ~4.9 nm^2 [2]
LA	4	---	~30	---	Footprint of 0.25 nm^2 by microscale thermogravimetric (TGA) analysis with quartz crystal microbalance (QCM) referred as µ-TGA [3]
PVP	1	---	~2-5	---	simulations [4]
	---	9-15	~20	~40	elemental analysis [1]
m-PEG-SH	5-10	---	~50	~5	hydrodynamic diameter and ζ-potential measurements [5]
	2.77 ± 0.43	---	~10	~5	Average value determined by total organic carbon (TOC) analysis and analytical ultracentrifugation (AUC) [6]
	0.8	---	62.5 ± 6	~10	TGA analysis [7]
BPEI	1.26	---	~9	~25	TGA analysis of covalently bound BPEI via 11-mercaptoundecanoid acid and amide bond [8]

"---" denotes not available or not existing values

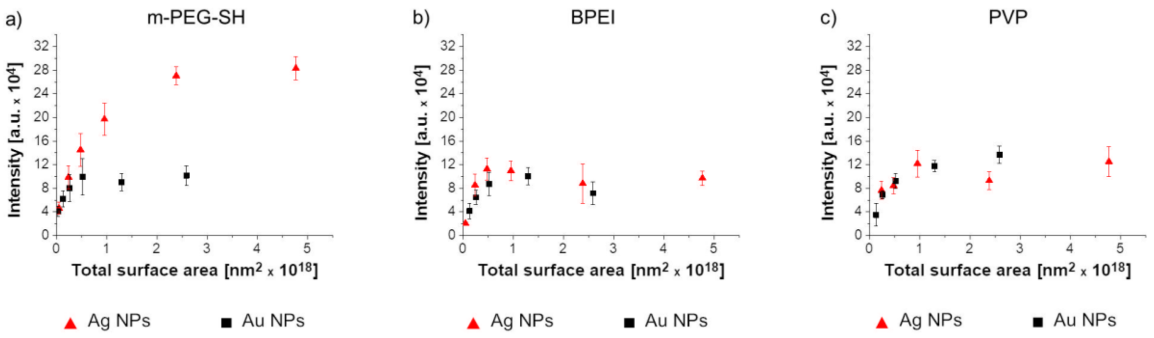

Fig. S42 Monomer signal intensity versus total surface area of 60 nm Au and Ag NPs capped with: a) m-PEG-SH, b) BPEI and c) PVP. Error bars were determined from three measurements of six spot replicates (n=18)

Estimation of capping agent concentrations using TOC measurements

Because the TOC measurements were all below LOD (Table S2) for the purified suspensions of Au NPs (50 mg/L) and Ag NPs (20 mg/L); these concentrations can only have the maximum capping agent concentration of 1 mg C/L. Here, we assume that 1 mg C/L is present in all capping agents. This value of 1 mg C/L has to be multiplied for Au NPs by the factor of: 1.91 for PVP, 1.83 for m-PEG-SH and 1.79 for BPEI. This factor is obtained by the division of the molar mass of the monomer by the molar mass of the monomer containing only carbon. For Ag NPs this factor is almost double: 3.82 for PVP, 3.67 for m-PEG-SH and 3.59 for BPEI because for the same Au NP mass concentration the number of Ag NPs is also double. This multiplication factor is needed to convert the mg C/L in mg/L for each polymer since TOC measures only the carbon content but the molecules contains also other elements. This obtained value corresponds for Au and Ag NPs for both 50 mg/L. Now, these corrected concentrations have to be divided by the molecular weight of the polymer (PVP = 40 kDa, m-PEG-SH = 5 kDa and BPEI = 25 kDa) to obtain the molar concentration of the capping agent for Au NPs and Ag NPs at 50 mg/L. From this starting value of 50 mg/L where the amount of capping agent is estimated, all analysed NP mass concentrations with LDI-ToF-MS can be converted to a capping agent concentration as shown in Table 3 in the main manuscript.

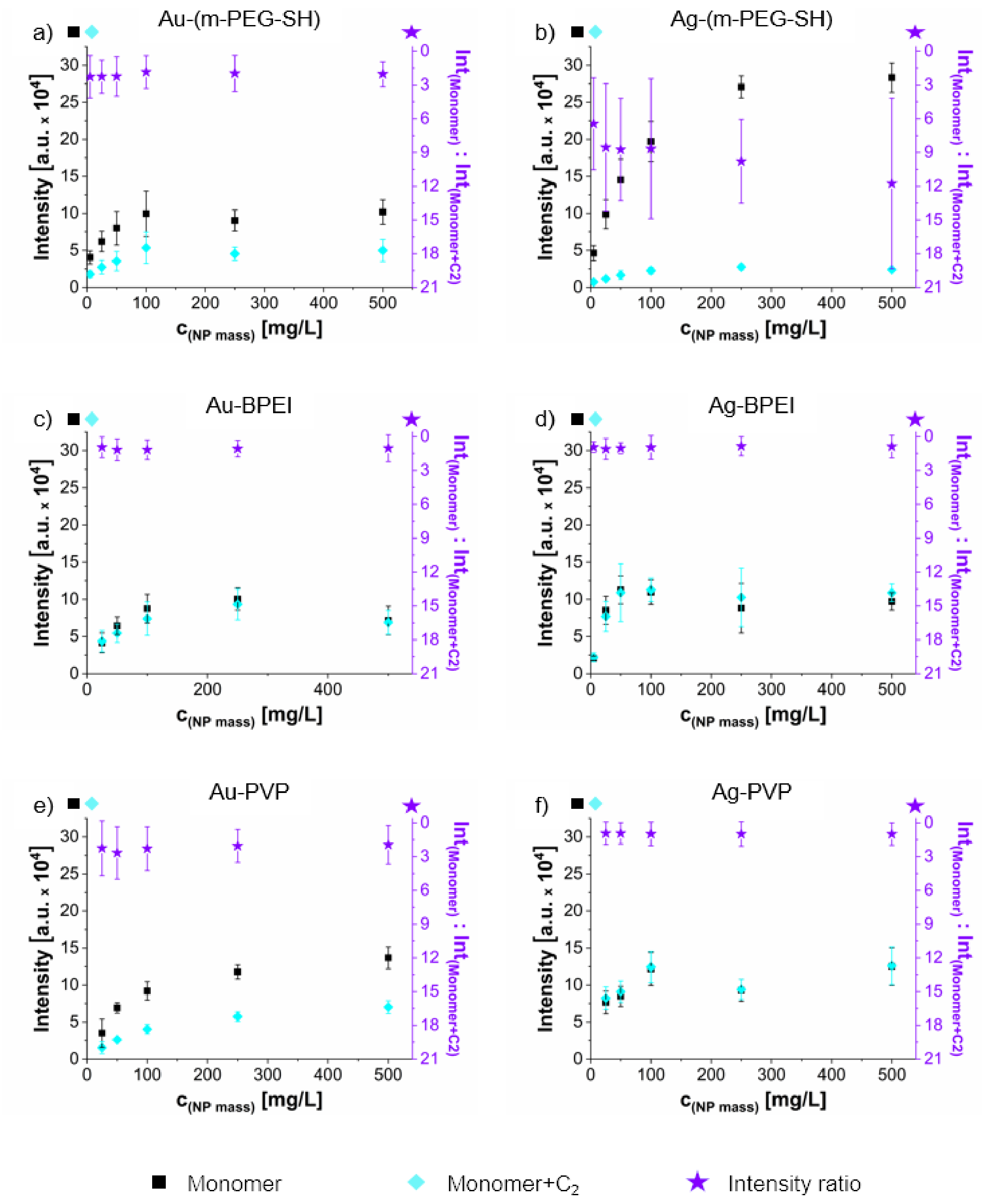

Fig. S43 Monomer and monomer+C_2 signal intensity versus NP mass concentration of the three polymeric capping agents on Au and Ag NPs: a) Au-(m-PEG-SH), b) Ag-(m-PEG-SH), c) Au-BPEI, d) Ag-BPEI, e) Au-PVP, f) Ag-PVP. Additionally, all ratios of the signal intensities between the monomer and the monomer+C_2 ions are plotted for each concentration in a second y-axis. Error bars were determined from three measurements of six spot replicates (*n*=18)

4 Size dependency of the signal intensity

Table S4 Calculated and reported values for the different sized m-PEG-SH capped Au NPs

Size [nm]	Total number of NPs in 50 mg/L	Surface area of 1 NP [nm^2]	Total surface area [nm^2]	Number of m-PEG-SH on 1 NP [7]	Total number of m-PEG-SH in 50 mg/L
30	$1.83 \cdot 10^{14}$	2826	$5.18 \cdot 10^{17}$	~916 ± 106	$1.68 \cdot 10^{17}$
60	$2.29 \cdot 10^{13}$	11,304	$2.59 \cdot 10^{17}$	~2572 ± 402	$5.89 \cdot 10^{16}$
100	$4.95 \cdot 10^{12}$	31,400	$1.55 \cdot 10^{17}$	~6778 ± 814	$3.36 \cdot 10^{16}$

The total number of m-PEG-SH in 50 mg/L for the different sizes were calculated by using the reported values of number of m-PEG-SH on 1 NP from Rahme et al. by multiplication with the calculated total number of NPs in 50 mg/L.

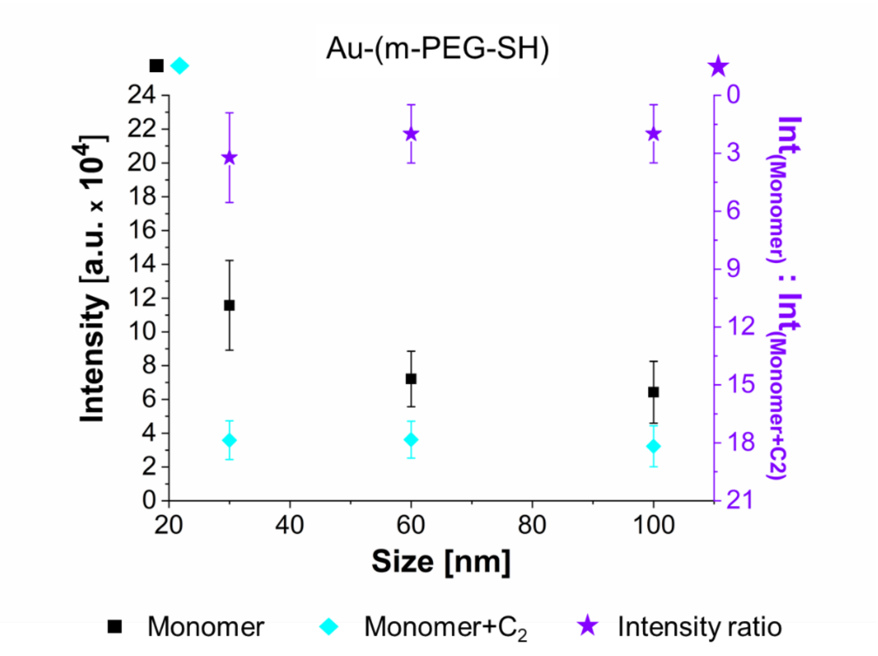

Fig. S44 Monomer and monomer+C_2 signal intensity versus NP size of 30, 60 and 100 nm for m-PEG-SH capped Au NPs. Additionally, all ratios of the signal intensities between the monomer and the monomer+C_2 ions are plotted for each concentration in a second y-axis. Error bars were determined from three measurements of six spot replicates (*n*=18)

5 Sorption of organic solutes onto capped NPs

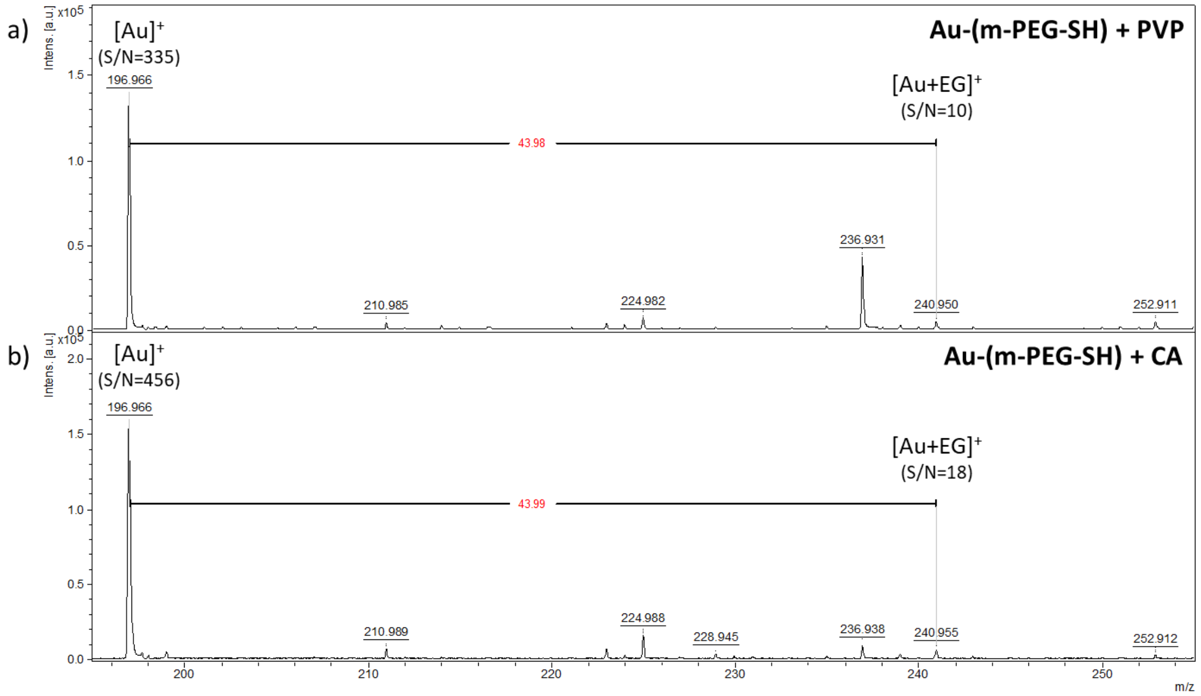

Fig. S45 Spectra of NP-solute interaction experiments of 60 nm m-PEG-SH capped Au NPs after interaction with a solution of a) 40 kDa PVP and b) CA showing the gold cluster ion together with the monomer of m-PEG-SH (theoretical monoisotopic neutral mass of C_2H_4O = 44.026 Da) as capping agent metal cluster at about *m/z* 240.96

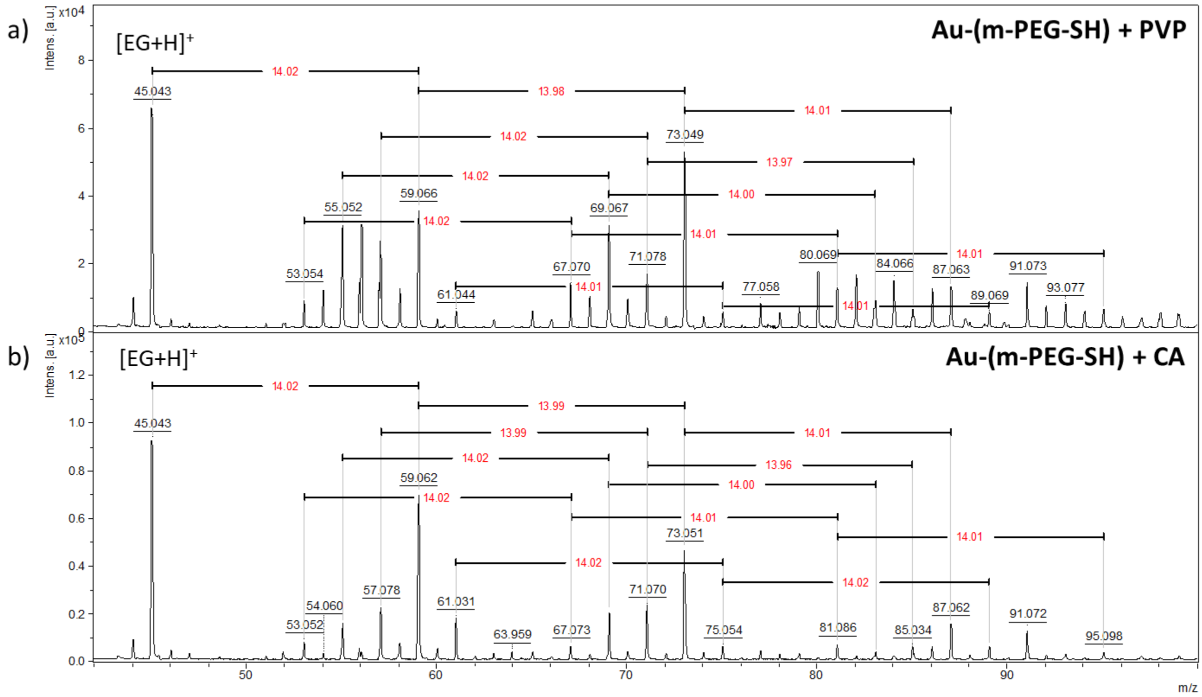

Fig. S46 Spectra of NP-solute interaction experiments of 60 nm m-PEG-SH capped Au NPs after interaction with a solution of a) 40 kDa PVP and b) CA showing capping agent fragment series of the CH_2 (theoretical monoisotopic neutral mass of CH_2 = 14.015 Da)

References

1. Rostek A, Mahl D, Epple M (2011) Chemical composition of surface-functionalized gold nanoparticles. J Nanoparticle Res 13:4809–4814 . https://doi.org/10.1007/s11051-011-0456-2

2. Wrigglesworth EG, Johnston JH (2017) The use of dual reductants in gold nanoparticle syntheses. RSC Adv 7:45757–45762 . https://doi.org/10.1039/c7ra07724f

3. Sebby KB, Mansfield E (2015) Determination of the surface density of polyethylene glycol on gold nanoparticles by use of microscale thermogravimetric analysis. Anal Bioanal Chem 407:2913–2922 . https://doi.org/10.1007/s00216-015-8520-x

4. Posel Z, Posocco P, Lísal M, Fermeglia M, Pricl S (2016) Highly grafted polystyrene/polyvinylpyridine polymer gold nanoparticles in a good solvent: Effects of chain length and composition. Soft Matter 12:3600–3611 . https://doi.org/10.1039/c5sm02867a

5. Perrault SD, Chan WCW (2010) Synthesis and Surface Modification of Highly Monodispersed, Spherical Gold Nanoparticles of 50-200 (vol 131, pg 17042, 2009). J Am Chem Soc 132:11824 . https://doi.org/10.1021/ja907069u

6. Benoit DN, Zhu H, Lilierose MH, Verm RA, Ali N, Morrison AN, Fortner JD, Avendano C, Colvin VL (2012) Measuring the grafting density of nanoparticles in solution by analytical ultracentrifugation and total organic carbon analysis. Anal Chem 84:9238–9245 . https://doi.org/10.1021/ac301980a

7. Rahme K, Chen L, Hobbs RG, Morris MA, O'Driscoll C, Holmes JD (2013) PEGylated gold nanoparticles: Polymer quantification as a function of PEG lengths and nanoparticle dimensions. RSC Adv 3:6085–6094 . https://doi.org/10.1039/c3ra22739a

8. Das J, Choi Y-J, Yasuda H, Han JW, Park C, Song H, Bae H, Kim J-H (2016) Efficient delivery of C/EBP beta gene into human mesenchymal stem cells via polyethylenimine-coated gold nanoparticles enhances adipogenic differentiation. Sci Rep 6:33784 . https://doi.org/10.1038/srep33784
